# ﻿Molecular phylogeny and morphology reveal four new species in Hymenochaetales and one new species in Cantharellales from Southwestern China

**DOI:** 10.3897/mycokeys.115.142433

**Published:** 2025-03-12

**Authors:** Jianling Zhang, Zirui Gu, Chunqin Zhou, Hongmin Zhou

**Affiliations:** 1 College of Forestry, Southwest Forestry University, Kunming 650224, China; 2 Yunnan Wumeng Mountains National Nature Reserve, Zhaotong 657000, China; 3 The Key Laboratory of Forest Resources Conservation and Utilization in the South-west Mountains of China Ministry of Education, Key Laboratory of National Forestry and Grassland Administration on Biodiversity Conservation in Southwest China, Yunnan Provincial Key Laboratory for Conservation and Utilization of In-forest Re-source, Southwest Forestry University, Kunming 650224, China

**Keywords:** Biodiversity, Cantharellales, Hymenochaetales, new taxa, wood-inhabiting fungi

## Abstract

Wood-decaying fungi represent a vital group of higher fungi that drive the cycling of matter and energy in forest ecosystems, and they have been the focus of thorough investigation. In this study, five new species, *viz. Botryobasidiumdaweishanense*, *Inonotussubglobisporum*, *Kneiffiellabubalina*, *Xylodongranulanoides*, and *X.granulans* from China, are described and illustrated based on the morphological characteristics and molecular phylogenetic analyses, in which the sequences of ITS+nLSU genes were used for the phylogenetic analyses by maximum likelihood and Bayesian inference methods. The phylogeny revealed that the *Botryobasidiumdaweishanense* groups with three taxa, *viz.*, *B.intertextum*, *B.leptocystidiatum*, and *B.subcoronatum*. *Inonotussubglobisporum* is closely related to *I.radiatus. Kneiffiellabubalina* clustered sister to *K.subalutacea*. *Xylodongranulanoides* and *X.granulans* have a close relationship with *X.bambusinus*, *X.fissuratus*, *X.subclavatus*, *X.montanus*, and *X.wenshanensis*. Additionally, *Xylodongranulanoides* and *X.granulans* clustered together. *Botryobasidiumdaweishanense* is characterized by an araneose hymenial surface, fusiform, and cyanophilous basidiospores (6.1–7.3 × 3.3–3.9 μm). *Inonotussubglobisporum* is characterized by perennial basidiomata with lateral stipes, polygon pores measuring 4–6 per mm, and subglobose, cyanophilous basidiospores (3.6–4.3 × 2.8–3.5 μm). *Kneiffiellabubalina* is characterized by cream basidiomata and cylindrical to slightly allantoid basidiospores (8.0–8.9 × 1.8–2.3 μm). *Xylodongranulanoides* is characterized by grandinioid hymenial surfaces, various cystidia, and broadly ellipsoid, thick-walled basidiospores (4.7–5.3 × 3.6–4.1 μm). *Xylodongranulans* is characterized by grandinioid hymenial surfaces, capitate and clavate cystidia, and broadly ellipsoid basidiospores (3.8–4.2 × 2.9–3.3 μm). Phylogenetic analysis based on internal transcribed spacer (ITS) and nuclear large subunit RNA (nLSU) shows that the four species are members of Hymenochaetales, and one belongs to Cantharellales. All five new species are compared with morphologically and phylogenetically closely related species. The present study contributes to understanding the species diversity, taxonomy, and phylogeny of macrofungi in Southwestern China.

## ﻿Introduction

Wood-inhabiting fungi thrive on various types of wood, including vigorous wood, dead wood, and fallen branches. They play a crucial role in degrading the lignin, cellulose, or hemicellulose present in wood, making them an important group of higher fungi (M’Barek et al. 2020; Runnel et al. 2021; Dong et al. 2023, 2024a). These fungi are the cornerstones of matter cycles and energy flows in forest ecosystems and play a vital ecological role in the regulation of carbon storage (Phookamsak et al. 2019; Ponce et al. 2023). A robust understanding of wood-fungi diversity is required to explain their rise to forest dominance; however, the classification of some taxa remains unknown.

The genus *Botryobasidium* Donk belongs to the family Botryobasidiaceae Jülich (Cantharellales, Basidiomycota) and is typified by *Botryobasidiumsubcoronatum* (Höhn. & Litsch.) Donk. Species of the corticioid *Botryobasidium* are saprobic fungi that cause white rot in forested areas (Langer et al. 2000b; Bondartseva and Zmitrovich 2023). They are commonly found on a range of hosts or substrates, from litter and fallen trunks to the stems of living trees, including macrophanerophytes (Langer et al. 2000a, 2000b; Hjortstam et al. 2005; Zhou et al. 2024a). Fungi in this genus have resupinate, smooth to grandinioid basidiomata; branched right-angled hyphae with or without clamp connections; short, cylindrical or subcylindrical to suburniform basidia with 2–8 sterigmata generally arranged in clusters; and narrow to globose, smooth to tuberculate or laciniate, and inamyloid basidiospores (Langer et al. 2000a; Bernicchia et al. 2010). According to the Index Fungorum (www.indexfungorum.org; accessed on 26 January 2025), the genus *Botryobasidium* has 116 specific registered names, with 101 species accepted worldwide (Dong et al. 2024b; Liu et al. 2024; Zhou et al. 2024a; Zhou et al. 2024b). Based on ITS data analysis, we demonstrated that the genus *Botryobasidium* formed a well-supported monophyletic group, as previously demonstrated by its micromorphological and ultrastructural characteristics (Langer and Langer 1998; Moncalvo et al. 2006; Zhou et al. 2024b).

Frey et al. (1977) established the order Hymenochaetales Oberw., using Hymenochaetaceae Donk as the type family (He et al. 2024). Hymenochaetales is a large order of Agaricomycetes with 14 families, 83 genera, and 1205 species, including wood-inhabiting and ectomycorrhizal fungi. These fungi have different kinds of basidiomata, such as polyporoid, stereoid, corticioid, hydnoid, coralloid, and agaricoid (Hibbett et al. 2014; He et al. 2024). Hymenochaetaceae includes species with brownish basidiomata and generative hyphae without clamp connections. This family includes six genera, namely, *Coltricia* Gray, *Cyclomyces* Kunze ex Fr., *Hydnochaete* Bres., *Hymenochaete* Lév., *Inonotus* P. Karst, and *Phellinus* Quél, laying the foundational framework for the classification of Hymenochaetaceae (Hibbett et al. 2014; Wijayawardene et al. 2022; He et al. 2024). However, the phylogeny of *Hymenochaete* is not well understood, and researchers have investigated the phylogenetic relationships of these genera (Wagner and Fischer 2002). Phylogenetic analyses of *Hymenochaete* and its allied genera have been performed, and the results have been verified by mycologists (Larsson et al. 2006; Dai 2010). Furthermore, a series of studies indicated that certain species have a high degree of host specificity (Dai 2010; Purahong et al. 2018; Wang et al. 2021, 2022; Wu et al. 2022; Zhao et al. 2023a; Zhou et al. 2023). The diversity of *Hymenochaete* fungi and the number of species recorded in China have also been extensively studied (Korotkin et al. 2018; Wu et al. 2022; Zhao et al. 2023a, 2024).

*Inonotus*, one of the largest genera within the Hymenochaetaceae family, is typified by *Inonotushispidus* (Bull.) P. Karst; it is primarily found as parasites or saprophytes on various types of wood (Wagner and Fischer 2002; Zhou et al. 2015). Fungi of this genus have annual to perennial, resupinate, effused reflexed or pileate, yellowish to brown, hispid, velutinate to rough or glabrous pilei; a brown pore surface; homogeneous, brown, corky context basidiomata; a monomitic hyphal system; and generative hyphae with simple septa. Hymenial setae may be present or absent; hyphoid setae are found in some species and are ellipsoid, hyaline to brownish, thick-walled basidiospores. Previous phylogenetic analyses indicated that *Inonotussensu lato* had polyphyletic origins, and four more narrowly defined genera, namely *Inocutis* Fiasson & Niemelä, *Inonotopsis* Parmasto, *Mensularia* Lázaro Ibiza, and *Onnia* P. Karst., were segregated from *Inonotus* (Wagner and Fischer 2002; Dai 2010). Meanwhile, two new genera were proposed (Zhou et al. 2016): *Sanghuangporus* Sheng H. Wu, L.W. Zhou & Y.C. Dai, and *Tropicoporus* L.W. Zhou, Y.C. Dai & Sheng H. Wu. *Inonotus* is a well-known species commonly used in traditional medicine to treat various ailments (Delgersaikhan et al. 2024; Yu et al. 2024). The genus produces white rot and exhibits a worldwide distribution (Wagner and Fischer 2002; Dai 2010), with 289 specific registered names and 128 species accepted worldwide (www.indexfungorum.org; accessed on 26 January 2025) (Wagner and Fischer 2002; Dai 2010; Zhou et al. 2016; Wu et al. 2022).

The genus *Kneiffiella* P. Karst belongs to the family Chaetoporellaceae Jülich (Hymenochaetales, Basidiomycota) and is typified by *Kneiffiellabarba-jovis* (Bull.) P. Karst. Fungi of this genus prefer dark microhabitats that slowly dry on tree trunks, leading to particularly severe wood decay, such as on hollow areas and undersides (Běťák et al. 2021). They have resupinate, smooth to grandinioid, coralloid, or irpicoid basidiomata; a white to brown or ochraceous hymenial surface; a monomitic to pseudodimitic hyphal system; generative hyphae with clamp connections; tubular and clavate cystidia; cylindrical to utriform, or barrel-like, basidia with four sterigmata; and cylindrical to ellipsoid or allantoid, inamyloid, and acyanophilous basidiospores (Wang et al. 2021). According to the Index Fungorum (www.indexfungorum.org; accessed on 26 January 2025), the genus *Kneiffiella* has 84 specific registered names, with 40 species accepted worldwide (Běťák et al. 2021; Wang et al. 2021; Viner et al. 2024).

The genus *Xylodon* (Pers.) Gray belongs to the family Schizoporaceae Jülich (Hymenochaetales, Basidiomycota) and is typified by *Xylodonquercinus* (Pers.) Gray (Bernicchia and Gorjón 2010). *Xylodon* is a large genus of corticioid fungi with a cosmopolitan distribution (Guan et al. 2023; Yurchenko et al. 2024; Zhang et al. 2024a). Species of *Xylodon* inhabit dead wood of various sizes, ranging from twigs of several millimeters in diameter to large fallen trunks, and they cause white rot (Greslebin and Rajchenberg 2000; Kotiranta and Saarenoksa 2000; Girometta et al. 2020; Guan et al. 2023). In some cases, basidiomata of *Xylodon* species appear on the living parts of trees (Yurchenko 2008) and non-woody plant remains, such as fern rachises (Kotiranta and Saarenoksa 2000), herb stems, fallen leaves (Viner et al. 2018), and dead polypore basidiomata (Viner et al. 2023). Fungi in this genus have resupinate or effuse, smooth basidiomata; a tuberculate, grandinioid, odontioid, coralloid, irpicoid, or poroid hymenial surface; a monomitic or dimitic hyphal system that is generative with clamp connections; different types of cystidia; utriform or suburniform basidia; and cylindrical to ellipsoid to globose basidiospores (Gray 1821; Bernicchia and Gorjón 2010; Zhang et al. 2024a). According to the Index Fungorum (www.indexfungorum.org; accessed on 26 January 2025), the genus *Xylodon* has 241 specific registered names, with 157 species accepted worldwide. Remarkably, new species have been described in this genus at an accelerated pace owing to advances in morphological taxonomy and molecular phylogeny (Luo et al. 2022; Qu et al. 2022; Guan et al. 2023; Yurchenko et al. 2024; Zhang et al. 2024a).

In addition, host specificity is essential for determining the taxonomy and phylogeny of Hymenochaetales, with various types identified in a series of studies, including angiosperms, gymnosperms, both angiosperms and gymnosperms, and bryophytes (Dai 2010; Zhao et al. 2024). Most species in Hymenochaetales are polyporous and corticioid fungi, whereas certain species, such as *Blasiphalia* Redhead, *Contumyces* Redhead, and *Rickenella* Raithelh, basidiomata in those taxa are agarics. Beyond morphological diversity, various trophic modes, including saprotrophs, parasites, and symbiotes (with both trees and moss), also exist within Hymenochaetales (Wang and Zhou 2024). Many new Hymenochaetales taxa have recently been described due to research on the diversity of wood-inhabiting fungi in Yunnan Province (Chen and Zhao 2020; Luo et al. 2021; Qu et al. 2022; Guan et al. 2023; Yuan and Zhao 2024; Zhang et al. 2024a). These studies provide an important foundation for further exploration of species diversity and taxonomic status within Hymenochaetales.

This study collected five new species of wood-inhabiting fungi from Yunnan Province, China. To clarify the taxonomic placement of these species, morphological and phylogenetic analyses based on the ITS and nLSU sequences were conducted to identify them as new species of *Botryobasidium*, *Inonotus*, *Kneiffiella*, and *Xylodon*. This study provides full descriptions, color photographs, detailed comparisons with closely related taxa, and phylogenetic placements of the five new species. New data have been added to the biodiversity research on the genera *Botryobasidium*, *Inonotus*, *Kneiffiella*, and *Xylodon*, further affirming the rich biodiversity of southwestern China.

## ﻿Materials and methods

### ﻿Sample collection and herbarium specimen preparation

The fresh basidiomata were collected on the fallen angiosperm branches from Yunnan Province, China, and collection information was recorded (Rathnayaka et al. 2024). The samples were photographed *in situ*, and fresh macroscopic details were recorded. Photographs were taken by a Nikon D7100 camera. All the photos were focus-stacked using Helicon Focus software. Macroscopic details were recorded, and the fruit bodies were transported to a field station where they were dried in an electric food dryer at 45 °C (Hu et al. 2022). Once dried, the specimens were sealed in an envelope and zip-lock plastic bags and labeled (Zhang et al. 2024b). The dried specimens were deposited in the herbarium of the Southwest Forestry University (SWFC), Kunming, Yunnan Province, China.

### ﻿Morphological studies

The macro-morphology was based on the fresh specimens, while the micro-morphology was studied based on dried specimens. The color terms in the description followed Anonymous (1969) and Petersen (1996). Micro-morphology was studied at magnifications of 1000 ×, using a Nikon Eclipse 80i microscope with phase contrast illumination. Melzer’s reagent (IKI), Cotton Blue (CB), and 5% potassium hydroxide (KOH) were used. Drawings were made with the aid of a drawing tube. In the text, further abbreviations were used: IKI− = non-dextrinoid and non-amyloid, IKI+ = amyloid, CB− = acyanophilous, CB+ = cyanophilous, L = mean basidiospore length (arithmetic average of all basidiospores), W = mean basidiospore width (arithmetic average of all basidiospores), Q = variation in the L/W ratios, n = the number of basidiospores measured.

### ﻿DNA Extraction, polymerase chain reaction, and sequencing genomic

The CTAB rapid plant genome extraction kit-DN14 (Aidlab Biotechnologies Co., Ltd., Beijing) was used to obtain DNA from dried specimens. PCR was performed according to the manufacturer’s instructions with some modifications (Yang et al. 2023). The nuclear ribosomal ITS region was amplified with the primers ITS5 and ITS4 (White et al. 1990). The nuclear ribosomal nLSU gene was amplified with the primers LR0R and LR7 (Vilgalys and Hester 1990; Rehner and Samuels 1994). The PCR procedure for ITS was as follows: initial denaturation at 95 °C for 3 min, followed by 35 cycles at 94 °C for 40 s, 54 °C for 45 s, and 72 °C for 1 min; and a final extension at 72 °C for 10 min. The PCR procedure for nLSU was as follows: initial denaturation at 94 °C for 1 min, followed by 35 cycles at 94 °C for 30 s, 50 °C for 1 min, and 72 °C for 1.5 min; and a final extension at 72 °C for 10 min (Zhou et al. 2024c). All newly generated sequences were submitted to GenBank and are listed in Table 1.

**Table 1.** Names, sample numbers, locations, references, and corresponding GenBank accession numbers of the taxa used in this study. [New species are shown in bold; * is shown as type material, holotype; — indicates sequence unavailability].

Species	Sample No.	GenBank No.		Country	References
		ITS	nLSU		
*Alloclavariapurpurea*	H6047663	MF319055	MF318905	Finland	Cho et al. 2024
*Alloclavariapurpurea*	M. Korhonen 10305	MF319044	MF318895	Finland	Cho et al. 2024
*Antrodiasubserpens*	Dai 13233	KP715309	KT968830	China	Chen and Cui 2015
*Athelodermamirabile*	TAA 169235	DQ873592	DQ873592	Estonia	Larsson et al. 2006
*Basidioradulumradula*	LWZ 20201017-62	ON063684	ON063884	China	Wang et al. 2023
*Blasiphaliapseudogrisella*	P. Hoijer 4118	MF319047	MF318898	Finland	Cho et al. 2024
*Blasiphaliapseudogrisella*	P. Hoijer 4393	MF319048	MF318899	Estonia	Cho et al. 2024
*Botryobasidiumacanthosporum*	Yuan18083*	PP229512	PP218361	China	Zhou et al. 2024c
*Botryobasidiumacanthosporum*	Yuan16326	PP229497	—	China	Zhou et al. 2024c
*Botryobasidiumasperulum*	RAS552	OR471090	OR470959	USA	Swenie et al. 2023
*Botryobasidiumasperulum*	FP102150	OR471075	OR47094	USA	Swenie et al. 2023
*Botryobasidiumaureum*	GEL 2910	AJ389783	—	Germany	Langer et al. 2000b
*Botryobasidiumbambusinum*	CLZhao29916	PQ539057	PQ539060	China	Dong et al. 2024b
*Botryobasidiumbambusinum*	CLZhao29936*	PQ539058	PQ539061	China	Dong et al. 2024b
*Botryobasidiumbotryosum*	KHL11081	AY463387	AY586638	Sweden	Larsson et al. 2004
*Botryobasidiumbotryosum*	AFTOL-ID 604	DQ267124	—	USA	Zhou et al. 2024c
*Botryobasidiumcandicans*	UC2022891	KP814227	—	USA	Vu et al. 2018
*Botryobasidiumcandicans*	UC2022944	KP814546	—	USA	Vu et al. 2018
*Botryobasidiumcandicans*	UC2022893	KP814200	—	USA	Vu et al. 2018
*Botryobasidiumconiferarum*	LWZ20171016-15	OR557262	—	China	Dong et al. 2024b
*Botryobasidiumconiferarum*	LWZ20210928-3*	OR557259	—	China	Dong et al. 2024b
*Botryobasidiumconspersum*	AFTOL-ID 1766	DQ911612	DQ521414	USA	Cao et al. 2021
*Botryobasidiumdaweishanense*	CLZhao40061	PQ373983	—	China	Present study
***Botryobasidiumdaweishanense***	**CLZhao40061**	**PQ373983**	—	**China**	**Present study**
***Botryobasidiumdaweishanense***	CLZhao40062*	**PQ373984**	**PQ373977**	**China**	**Present study**
*Botryobasidiumgossypirubiginosum*	CLZhao 26052*	OR668924	OR708665	China	Zhou et al. 2024b
*Botryobasidiumincanum*	CLZhao 26697*	OR668923	OR708664	China	Zhou et al. 2024b
*Botryobasidiumindicum*	NFCCI4480	NR171230	—	India	Zhou et al. 2024b
*Botryobasidiumindicum*	AMH:10054	MK391496	—	India	Zhou et al. 2024b
*Botryobasidiumindicum*	hr5326	OP806032	—	China	Zhou et al. 2024b
*Botryobasidiumindicum*	CLZhao21791	ON406471	—	China	Zhou et al. 2024b
*Botryobasidiumindicum*	AMH:10054	MK391496	MK391493	India	Zhou et al. 2024b
*Botryobasidiumintertextum*	UC2022959	KP814540	—	North American	Zhou et al. 2024b
Botryobasidium laeve	RAS762	OR471128	—	USA	Swenie et al. 2023
*Botryobasidiumleptocystidiatum*	Yuan17548	PP209211	—	China	Zhou et al. 2024c
*Botryobasidiumleptocystidiatum*	Yuan17708*	PP209197	—	China	Zhou et al. 2024c
*Botryobasidiumrobustius*	CBS:945.69	MH859491	MH871272	Czech Republic	Fukami et al. 2010
*Botryobasidiumsimile*	RAS793	OR471146	—	USA	Swenie et al. 2023
*Botryobasidiumsimile*	RAS794	OR471147	—	USA	Swenie et al. 2023
*Botryobasidiumsimile*	GEL2348	KP171641	DQ898730	Canada	Cao et al. 2021
*Botryobasidiumsubcoronatum*	RAS620 SV1	OR471110	—	USA	Swenie et al. 2023
*Botryobasidiumsubcoronatum*	RAS789	OR471144	—	USA	Swenie et al. 2023
*Botryobasidiumsubcoronatum*	FP101015	OR471077	—	USA	Swenie et al. 2023
*Botryobasidiumsubcoronatum*	FP151108	OR471078	—	USA	Swenie et al. 2023
*Botryobasidiumsubcoronatum*	AFTOL-ID 614	DQ200924	AY647212	USA	Cao et al. 2021
*Botryobasidiumsubovalibasidium*	Yuan18179*	PP209196	—	China	Zhou et al. 2024c
*Botryobasidiumsubovalibasidium*	Yuan16439	PP209199	—	China	Zhou et al. 2024c
*Botryobasidiumtubulicystidium*	DK14139	OL436769	—	USA	Zhou et al. 2024b
*Botryobasidiumyunnanense*	CLZhao24877*	OR668925	—	China	Zhou et al. 2024b
*Bridgeoporussinensis*	Cui 10013	KY131832	KY131891	China	Cho et al. 2024
*Bryoclavulaphycophila*	TNS F-79667	NR169921	LC508118	Japan	Masumoto and Degawa 2020
*Bryoclavulaphycophila*	S-287-FB3	LC544109	LC544110	Japan	Masumoto and Degawa 2020
*Bryopistillariasagittiformis*	IO.14.164	MT232349	MT232303	Sweden	Olariaga et al. 2020
*Burgellalutea*	Etayo 27623	KC336076	KC336075	Bolivia	Diederich et al. 2014
*Burgoaverzuoliana*	CBS 131.38	NR145334	NG058614	Japan	Cao et al. 2021
*Cantharellopsisprescotii*	H6059300	MF319051	MF318903	Finland	Cho et al. 2024
*Cantharellusalbidolutescens*	BB 08.070*	KF981365	KF294646	Madagascar	Cao et al. 2021
*Cantharellusalborufescens*	AH44223	KR677493	KR677531	Spain	Olariaga et al. 2015
*Cantharellusalborufescens*	BB 12.075	KX907209	KX929161	Switzerland	Olariaga et al. 2017
*Cantharellusambohitantelyensis*	BB 08.336*	KF981366	KF294656	Madagascar	Cao et al. 2021
*Cantharellusamethysteus*	BB 07.284	JN944020	KF294639	Slovakia	Olariaga et al. 2017
*Cantharellusamethysteus*	AH44796*	KR677512	KR677550	Spain	Olariaga et al. 2015
*Cantharellusanzutake*	TNS-F-61925*	LC085359	LC085415	Japan	Cao et al. 2021
*Cantharelluscalifornicus*	OSC 122878 *	KX828768	KX828795	USA	Olariaga et al. 2017
*Cantharelluscascadensis*	OSC 75908	AY041181	AY041160	USA	Olariaga et al. 2015
*Cantharelluschicagoensis*	PRL8916	KP639201	KP639218	USA	Leacock et al. 2016
*Cantharelluschicagoensis*	PRL8332	KP639200	KP639214	USA	Leacock et al. 2016
*Cantharelluscibarius*	BIO-Fungi 10986*	KR677501	KR677539	Sweden	Olariaga et al. 2015
*Cantharelluscyphelloides*	TNS:F-61721*	NR154853	NG059027	Japan	Suhara and Kurogi 2015
*Cantharellusdecolorans*	469/BB 08.278	NR154788	KF294654	Madagascar	Olariaga et al. 2017
*Cantharellusferruginascens*	BIO-Fungi 11700	KR677486	KR677524	Spain	Olariaga et al. 2015
*Cantharellushygrophorus*	BB 08.196*	KF981368	KF294650	Madagascar	Cao et al. 2021
*Cantharelluslewisii*	BB 07.003*	JN944021	JN940597	USA	Olariaga et al. 2015
*Cantharelluspallens*	BB 09.409	KX929162	KX907215	Italy	Olariaga et al. 2017
*Cantharellusromagnesianus*	AH44218	KX828784	KX828807	Spain	Olariaga et al. 2017
*Cantharellusroseocanus*	DAOM220723	KX828787	KX828810	Canada	Olariaga et al. 2017
*Cantharellussebosus*	BB 08.234*	NR154789	KF294652	Madagascar	Cao et al. 2021
*Cantharellussebosus*	BB 08.162	KF981371	KF294649	Madagascar	Cao et al. 2021
*Cantharellussubalbidus*	OSC 75937	AY041179	AY041149	USA	Dunham et al 2003
*Cantharellussubincarnatus*	BB 06.096	KF981372	KF294602	Madagascar	Cao et al. 2021
*Cantharellussubminor*	Yuan 13917*	MW980545	MW979522	China	Cao et al. 2021
*Cantharellussubminor*	Yuan 13925	MW980546	MW979523	China	Cao et al. 2021
*Cantharellussubminor*	Yuan 13926	MW980547	MW979524	China	Cao et al. 2021
*Cantharellustabernensis*	BB 07.064	JN944012	JN940608	USA	Olariaga et al. 2015
*Cantharellustabernensis*	BB 07.040	JN944013	JN940609	USA	Olariaga et al. 2015
*Cantharellustenuithrix*	BB 07.125*	JN944017	JN940600	USA	Olariaga et al. 2017
*Cantharellusvaginatus*	HKAS55730*	HQ416692	HM594681	China	Cao et al. 2021
*Cantharellusyunnanensis*	Yuan 14539	MW980541	MW979514	China	Cao et al. 2021
*Cantharellusyunnanensis*	Yuan 14636	MW980542	MW979515	China	Cao et al. 2021
*Ceratobasidiumglobisporum*	CBS 569.83	DQ278942	MH873365	Australia	Cao et al. 2021
*Ceratobasidium* sp.	CAG6	AF354083	AF354083	USA	Gonzalez et al. 2001
*Ceratorhizahydrophila*	E14504F	MT381956	MT381951	Ecuador	Adaku et al. 2020
*Cerioporussquamosus*	Cui 10595	KU189778	KU189809	China	Zhou et al. 2016
*Clavulinacerebriformis*	MCA4022*	NR121504	JN228222	Guyana	Cao et al. 2021
*Clavulinacf.cristata*	MES426	JN228225	JN228225	China	Cho et al. 2024
*Clavulinacinereoglebosa*	TH8561	JN228218	JN228232	Guyana	Cho et al. 2024
*Clavulinacristata*	JKU8	JN228227	JN228227	USA	Cho et al. 2024
*Clavulina* sp.	MB03-034	DQ202266	AY745694	USA	Cao et al. 2021
*Coltriciaabieticola*	Cui 10321	KX364785	—	China	Unpublished
*Coniferiporiaqilianensis*	Yuan 6424	NR_158318	NG_060411	China	Wu et al. 2022
*Coniferiporiaweirii*	FP-134599-SP	MT420695	MT416461	China	Cho et al. 2024
*Contumycesrosellus*	MGW1462	MF319059	MF318912	USA	Cho et al. 2024
*Cotylidiafbrae*	FM639	NR_176148	NG_088193	China	Cho et al. 2024
*Cotylidia* sp.	AFTOL-700	AY854079	AY629317	USA	Cho et al. 2024
*Craterellusatratoides*	TH8473	JQ915103	JQ915129	Guyana	Wilson et al. 2012
*Craterellusatratoides*	TH9232*	JQ915111	NG042660	Guyana	Wilson et al. 2012
*Craterellusatratus*	MCA1070	JQ915092	JQ915118	Guyana	Wilson et al. 2012
*Craterellusatratus*	MCA990	JQ915100	JQ915126	Guyana	Wilson et al. 2012
*Craterellusatrobrunneolus*	Yuan 13878	MN902353	MN894058	China	Wilson et al. 2012
*Craterellusbadiogriseus*	Yuan 14776*	MW980548	MW979532	China	Cao et al. 2021
*Craterellusbadiogriseus*	Yuan 14779	MW980549	MW979533	China	Cao et al. 2021
*Craterelluscinereoﬁmbriatus*	TH8999	JQ915104	JQ915130	Guyana	Wilson et al. 2012
*Craterelluscinereoﬁmbriatus*	TH9075*	JQ915105	JQ915131	Guyana	Wilson et al. 2012
*Craterelluscroceialbus*	Yuan 14623*	MW980572	MW979529	China	Cao et al. 2021
*Craterelluscroceialbus*	Yuan 14647	MW980573	MW979530	China	Cao et al. 2021
*Craterellusexcelsus*	TH7515	JQ915101	JQ915127	Guyana	Wilson et al. 2012
*Craterellusexcelsus*	TH8235*	JQ915102	JQ915128	Guyana	Wilson et al. 2012
*Craterellusfallax*	AFTOL-ID 286	DQ205680	AY700188	USA	Cao et al. 2021
*Craterellusindicus*	PUN 3884*	NR119831	NG060387	India	Kumari et al. 2012
*Craterellusluteus*	GDGM48105*	MG727896	MG701171	China	Zhong et al. 2018
*Craterellusluteus*	GDGM46432	MG727897	MG727898	China	Zhong et al. 2018
*Craterellusmacrosporus*	Yuan 14782	MW980574	MW979531	China	Cao et al. 2021
*Craterellusolivaceoluteus*	MCA3186	JQ915098	JQ915124	Guyana	Wilson et al. 2012
*Craterellusolivaceoluteus*	TH9205*	JQ915109	JQ915135	Guyana	Wilson et al. 2012
*Craterellusparvogriseus*	CAL 1533*	MF421099	MF421098	India	Cao et al. 2021
*Craterelluspleurotoides*	MCA3124	JQ915097	JQ915123	Guyana	Wilson et al. 2012
*Craterelluspleurotoides*	TH9220	JQ915110	JQ915136	Guyana	Wilson et al. 2012
*Craterellusstrigosus*	MCA1750	JQ915094	JQ915120	Guyana	Wilson et al. 2012
*Craterellusstrigosus*	TH9204*	JQ915108	JQ915134	Guyana	Wilson et al. 2012
*Cylindrosporusflavidus*	Dai 13213	KP875564	KP875561	China	Wu et al. 2022
*Dacrymycesaustralis*	CBS:196.63	MH858261	MH869866	USA	Cao et al. 2021
*Fasciodontiabrasiliensis*	MSK-F 7245a*	MK575201	MK598734	Brazil	Yurchenko et al. 2020a
*Fasciodontiabugellensis*	KAS-FD 10705a	MK575203	MK598735	France	Yurchenko et al. 2020a
*Fasciodontiayunnanensis*	CLZhao 6280	MK811275	MZ146327	China	Luo and Zhao 2021
*Fibrodontiagossypina*	AFTOL-ID 599	DQ249274	—	USA	Unpublished
*Flaviporellussplitgerberi*	JV 1908/6	MZ484525	MZ437386	French Guiana	Wu et al. 2022
*Fomitiporellainermis*	JV 0509/57K	KX181305	KX181346	USA	Ji et al. 2018
*Fomitiporellasubinermis*	Dai 15114	KX181308	KX181344	China	Ji et al. 2018
*Fomitiporellavietnamensis*	Dai 18377*	NR_158436	NG_060441	Vietnam	Ji et al. 2018
*Fomitiporialangloisii*	MUCL 46375	EF429242	EF429225	USA	Decock et al. 2007
*Fomitopsispinicola*	AFTOL 770	AY854083	AY684164	USA	Unpublisheded
*Fulvifomesindicus*	Yuan 5932	KC879261	JX866777	China	Wu et al. 2022
*Fuscoporiaferruginosa*	JV0408/28	KX961103	KY189103	China	Chen and Yuan 2017
*Globuliciumhiemale*	Hjm 19007	DQ873595	DQ873595	Sweden	Larsson et al. 2006
*Gyroflexusbrevibasidiatus*	IO.14.230	MT232351	MT232305	Sweden	Olariaga et al. 2020
*Hastodontiahalonata*	HHB-17058	MK575207	MK598738	Mexico	Yurchenko et al. 2020a
*Heterobasidionannosum*	06129/6	KJ583211	KJ583225	China	Chen et al. 2014
*Hirschioporusabietinus*	Cui 2667	OQ449096	OQ449033	China	Cho et al. 2024
*Hirschioporusabietinus*	Dai 23760	OQ449039	OQ449034	China	Cho et al. 2024
*Hirschioporusacontextus*	Dai 19097	OQ449140	OQ449199	China	Cho et al. 2024
*Hirschioporusacontextus*	Dai 23793*	OQ449141	OQ449200	China	Cho et al. 2024
*Hirschioporusbeijingensis*	Dai 18907	OQ449142	OQ449201	China	Cho et al. 2024
*Hirschioporusbeijingensis*	Dai 23704*	OQ449143	OQ449202	China	Cho et al. 2024
*Hirschioporuschinensis*	Dai 20264	OQ449101	OQ449204	China	Cho et al. 2024
*Hirschioporuschinensis*	Dai 23048	OQ437349	OQ438002	China	Cho et al. 2024
*Hirschioporusfuscoviolaceus*	Dai 20988	OQ437357	OQ438006	Belarus	Cho et al. 2024
*Hirschioporusfuscoviolaceus*	Cui 10439	OQ437361	OQ438010	China	Cho et al. 2024
*Hirschioporusfuscoviolaceus*	KUC20131001-03	KJ668436	KJ668288	South Korea	Jang et al. 2015
*Hirschioporuspubescens*	Dai 17064*	OQ437377	OQ438019	China	Cho et al. 2024
*Hirschioporuspubescens*	Dai 23710	OQ512026	OQ449059	China	Cho et al. 2024
*Hirschioporustianschanicus*	Dai 19067*	OQ448960	OQ449067	China	Cho et al. 2024
*Hirschioporustianschanicus*	Dai 19064	OQ437386	OQ449066	China	Cho et al. 2024
*Holtermanniellawattica*	CBS 9496*	NR138371	NG058307	Antarctica	Cao et al. 2021
*Hydnoporiaolivacea*	Dai 12789	KT828678	KT828679	USA	Yang et al. 2016
*Hydnoporiatabacina*	LWZ 20210924-26a	ON063651	ON063851	China	Wang et al. 2023
*Hydnumalbomagnum*	AFTOL-ID 471	DQ218305	AY700199	USA	Cao et al. 2021
*Hydnumalbomagnum*	Wei 10194	MW980550	MW979536	China	Cao et al. 2021
*Hydnumalbomagnum*	Wei 10247	MW980551	MW979537	China	Cao et al. 2021
*Hydnumberkeleyanum*	CAL 1656*	NR158533	NG070500	India	Cao et al. 2021
*Hydnumberkeleyanum*	HKAS77834	KU612525	KU612667	China	Cao et al. 2021
*Hydnumberkeleyanum*	Wei 10375	MW980552	MW979538	China	Cao et al. 2021
*Hydnumbrevispinum*	Wei 10214*	MW980578	MW979559	China	Cao et al. 2021
*Hydnumcremeoalbum*	HKAS92345	KU612619	KU612676	China	Cao et al. 2021
*Hydnumellipsosporum*	FD3281	KX086215	KX086217	Switzer	Cao et al. 2021
*Hydnumﬂabellatum*	Yuan 14708*	MW980575	MW979556	China	Cao et al. 2021
*Hydnumﬂavidocanum*	Yuan 13903a*	MW980559	MW979545	China	Cao et al. 2021
*Hydnumﬂavidocanum*	Yuan 13900a	MW980560	MW979546	China	Cao et al. 2021
*Hydnumjussii*	Yuan 14008	MW980553	MW979539	China	Cao et al. 2021
*Hydnumjussii*	Yuan 14009	MW980554	MW979540	China	Cao et al. 2021
*Hydnumlongibasidium*	Wei 10383*	MW980556	MW979541	China	Cao et al. 2021
*Hydnumlongibasidium*	Wei 10367	MW980555	MW979542	China	Cao et al. 2021
*Hydnummagnorufescens*	voucher 161209	KU612549	KU612669	China	Cao et al. 2021
*Hydnumminum*	N.K.Zeng2819	KY407533	KY407528	China	An et al. 2017
*Hydnumminum*	Wei 10252	MW980557	MW979543	China	Cao et al. 2021
*Hydnumminum*	Wei 10260	MW980558	MW979544	China	Cao et al. 2021
*Hydnumpallidocroceum*	Yuan 14023*	MW980568	MW979554	China	Cao et al. 2021
*Hydnumpallidocroceum*	Yuan 14017	MW980569	MW979555	China	Cao et al. 2021
*Hydnumpallidomarginatum*	Yuan 13928a*	MW980566	MW979552	China	Cao et al. 2021
*Hydnumpallidomarginatum*	Yuan 13940a	MW980567	MW979553	China	Cao et al. 2021
*Hydnumrepandum*	H 6003710*	KX388650	—	Finland	Dong et al. 2024b
*Hydnumsphaericum*	Wei 10243*	MW980563	MW979549	China	Cao et al. 2021
*Hydnumsubolympicum*	F1188765	KU612599	KU612653	USA	Cao et al. 2021
*Hydnumsubrufescens*	F1188749	KU612535	KU612663	USA	Cao et al. 2021
*Hydnumtangerinum*	Wei 10245*	MW980580	MW979561	China	Cao et al. 2021
*Hydnumtenuistipitum*	Wei 10410*	MW980576	MW979557	China	Cao et al. 2021
*Hydnumventricosum*	Yuan 14536*	MW980561	MW979547	China	Cao et al. 2021
*Hymenochaeterubiginosa*	He 1049	JQ716407	JQ279667	China	He and Li 2012
*Hyphodontiaabieticola*	GEL2924	DQ340332	—	Germany	Unpublished
*Hyphodontiaabieticola*	KHL 12498	DQ873601	—	Sweden	Unpublished
*Hyphodontiaalutaria*	GEL3183	DQ340318	—	Germany	Zhang et al. 2024a
*Hyphodontiaalutaria*	KHL 11889	DQ873603	—	Sweden	Unpublished
*Hyphodontiaalutaria*	KHL 11978	EU118631	—	Norway	Unpublished
*Hyphodontiaargut*	Wu 0806-44	JN571548	—	China	Unpublished
*Hyphodontiaarguta*	KHL11938	EU118632	—	Sweden	Larsson 2007
*Hyphodontiaborbonica*	FR-0219441*	KR349240	—	France	Unpublished
*Hyphodontiaborbonica*	FR-0219444	KR349241	—	France	Unpublished
*Hyphodontiacurvispora*	1591a	DQ873615	—	Finland	Unpublished
*Hyphodontiahastata*	GEL2143	DQ340323	—	Germany	Unpublished
*Hyphodontiahastata*	GEL3124	DQ340311	—	Germany	Unpublished
*Hyphodontiamongolica*	Cui 13240	KY290985	—	China	Unpublished
*Hyphodontiamongolica*	Cui 13239*	KY290984	—	China	Unpublished
*Hyphodontiapachyspora*	LWZ20180905-6	MT319425	—	China	Wang et al. 2021
*Hyphodontiapachyspora*	LWZ 20170908-5*	MT319426	MT319160	China	Wang et al. 2021
*Hyphodontiapallidula*	KAS-GEL2097	DQ340317	—	Germany	Zhang et al. 2024a
*Hyphodontia* sp.	LWZ20180511-2	MT319418	—	China	Wang et al. 2021
*Hyphodontia* sp.	LWZ20170814-15	MT319417	—	China	Wang et al. 2021
*Hyphodontiasubdetritica*	TU114869	OP620786	—	France	Viner et al. 2023
*Hyphodontiasubdetritica*	FR-0261087	KY081794	—	France	Unpublished
*Hyphodontiasubdetritica*	FR-0261085	KY081793	—	France	Unpublished
*Hyphodontiatropica*	ICMP 13835	AF145586	—	China	Viner et al. 2023
*Hyphodontiatropica*	ICMP 13837	AF145587	—	China	Unpublished
*Hyphodontiawongiae*	LWZ20180417-16	MT319416	—	China	Wang et al. 2021
*Hyphodontiawongiae*	LWZ20180417-8	MT319415	—	China	Wang et al. 2021
*Hyphodontiawongiae*	LWZ20180414-16*	MT319414	—	China	Wang et al. 2021
*Hyphodontiazhixiangii*	LWZ 20170818-13	MT319420	MT319151	China	Wang et al. 2021
*Hyphodontiazhixiangii*	LWZ20180903-5	MT319423	—	China	Luo et al. 2022
*Hyphodontiazhixiangii*	LWZ20170820-31	MT319422	—	China	Wang et al. 2021
*Hyphodontiazhixiangii*	LWZ20170820-27	MT319421	—	China	Wang et al. 2021
*Hyphodontiazhixiangii*	LWZ20180903-9	MT319424	—	China	Wang et al. 2021
*Hyphodontiazhixiangii*	LWZ 20160909-8	KY440397	—	China	Wang et al. 2021
*Hyphodontiazhixiangii*	LWZ 20160909-4*	KY440396	—	China	Wang et al. 2021
*Hyphodontiazhixiangii*	LWZ 20160909-9	KY440398	—	China	Wang et al. 2021
*Hyphodontiazhixiangii*	LWZ20180904-12	MT319419	—	China	Wang et al. 2021
*Inonotopsissubiculosa*	Dai 14799	KU598212	KU598217	China	Unpublished
*Inonotusandersonii*	SFCC 50025	AY558599	AY558599	South Korea	Unpublished
*Inonotusandersonii*	JV 1209/66	KF446594	—	China	Unpublished
*Inonotusandersonii*	CS-65-92-14-B	OQ539568	—	Indiana	Unpublished
*Inonotusboninensis*	Dai 18868	MZ484601	—	Australia	Wu et al. 2022
*Inonotuscostaricensis*	JV 1511/171J	MZ484602	—	USA	Wu et al. 2022
*Inonotuscuticularis*	JV 0609/22	MN318442	MN318442	Czech Republic	Wu et al. 2022
*Inonotusdentiporus*	MUCL 4227	MZ484608	—	Brazil	Wu et al. 2022
*Inonotusgriseus*	LWZ 20130810-20*	KM434333	—	China	Zhou and Wang 2015
*Inonotushenanensis*	Dai 13157*	KP030783	KX832918	China	Zhou and Wang 2015
*Inonotushenanensis*	Dai 12220	MZ484603	—	China	Wu et al. 2022
*Inonotushenanensis*	Dai 13157	KX674581	—	China	Wu et al. 2022
*Inonotushispidus*	Cui 11932	MZ484604	—	China	Wu et al. 2022
*Inonotushispidus*	S45	EU282482	—	Spain	Wu et al. 2022
*Inonotuskrawtzewii*	JV 8709/35	KF446598	—	China	Wu et al. 2022
*Inonotuskrawtzewii*	PRM 607951	KF446600	—	China	Wu et al. 2022
*Inonotuslatemarginatus*	Dai 9758*	KP030784	—	China	Zhou and Wang 2015
*Inonotusmicantissimus*	URM90186	MG576057	—	Brazil	Wu et al. 2022
*Inonotusnidus-pici*	JV01076	MN318440	—	Czech Republic	Wu et al. 2022
*Inonotusniveomarginatus*	Dai 12318*	KC456245	—	China	Yu et al. 2013
*Inonotusobliquus*	JV 0408/36	KF446605	—	China	Wu et al. 2022
*Inonotusobliquus*	Dai 10715	MZ484606	—	Finland	Wu et al. 2022
*Inonotusplorans*	Yang 52	MZ484607	—	China	Wu et al. 2022
*Inonotusportoricensis*	JV 1504/121	MN318447	MN318447	Costa Rica	Unpublished
*Inonotuspseudoglomeratus*	JV1707/15J	MN318437	—	Costa Rica	Wu et al. 2022
*Inonotusquercustris*	193	AY072026	—	Argentina	Gottlieb et al. 2002
*Inonotusradiatus*	SAT-10-240-02	MT955156	MT955156	USA	Unpublished
*Inonotusradiatus*	HBAU15722	MW862276	—	China	Unpublished
*Inonotusradiatus*	HMAS 292289	OR237100	—	China	Unpublished
*Inonotusradiatus*	HMAS 281802	OR236984	—	China	Unpublished
*Inonotusradiatus*	MQ18R132-QFB30648	MN992228	—	Canada	Unpublished
*Inonotusradiatus*	DM1160	MT644872	—	Denmark	Unpublished
*Inonotusradiatus*	Cui 10321	KX364785	—	China	Unpublished
*Inonotusrickii*	Dai 12996	KC479128	—	China	Wu et al. 2022
*Inonotusrickii*	JV 1612/21J	MZ484609	—	China	Wu et al. 2022
*Inonotussetulosocroceus*	STE-U7801	KP279294	—	South Korea	Wu et al. 2022
***Inonotussubglobisporum***	**CLZhao 8331**	**PQ373985**	—	**China**	**Present study**
***Inonotussubglobisporum***	**CLZhao 8387**	**PQ373986**	**PQ373978**	**China**	**Present study**
***Inonotussubglobisporum***	**CLZhao 8433**	**PQ373987**	**PQ373979**	**China**	**Present study**
***Inonotussubglobisporum***	**CLZhao 8453**	**PQ373988**	—	**China**	**Present study**
***Inonotussubglobisporum***	**CLZhao 8500**	**PQ373989**	**PQ373980**	**China**	**Present study**
***Inonotussubglobisporum***	**CLZhao 8678**	**PQ373990**	—	**China**	**Present study**
***Inonotussubglobisporum***	**CLZhao 8737**	**PQ373991**	—	**China**	**Present study**
***Inonotussubglobisporum***	CLZhao 8765*	**PQ373992**	—	**China**	**Present study**
***Inonotussubglobisporum***	**CLZhao 8789**	**PQ373993**	—	**China**	**Present study**
*Inonotussubradiatus*	Dai 20201*	MZ484610	—	China	Wu et al. 2022
*Inonotustenuicontextus*	Yuan 5526*	NR_119969	—	China	Zhou and Qin 2011
*Inonotusulmicola*	H 6012614	KF446606	—	China	Wu et al. 2022
*Inonotusungulatus*	Dai 18864	MZ484611	—	Australia	Wu et al. 2022
*Inonotusvieinamensis*	Dai 18310	MZ484613	—	Viet Nam	Wu et al. 2022
*Inonotusvieinamensis*	Dai 18288*	MZ484612	—	Viet Nam	Zhou and Qin 2011
*Inonotusvitis*	OC 1*	MN108118	MN113944	USA	Brown et al .2019
*Kneiffiellabarba-jovis*	KHL 11730	DQ873609	DQ873610	Sweden	Unpublished
*Kneiffiellasubglobosa*	LWZ 20180416-6	MT319413	MT319145	China	Wang et al. 2021
*Kneiffiellaabdita*	Miettinen 22165	ON188809	ON188809	Finland	Viner et al. 2024
*Kneiffiellaabieticola*	KHL 12498 (GB)	DQ873601	—	Sweden	Larsson et al. 2006
*Kneiffiellaalienata*	CBS 127219	MH864327	—	USA	Vu et al. 2018
*Kneiffiellaaltaica*	PRM 956491	OM971678	—	Czech Republic	Langer et al. 2022
*Kneiffiellaaltaica*	PRM 956489	OM971676	—	Czech Republic	Langer et al. 2022
*Kneiffiellaaltaica*	PRM 956490	OM971677	—	Czech Republic	Langer et al. 2022
*Kneiffiellaaltaica*	PRM 953309	OM971675	—	Czech Republic	Langer et al. 2022
*Kneiffiellaalutacea*	KAS-GEL 2284	DQ340340	—	Germany	Yurchenko et al. 2020a
*Kneiffiellaalutacea*	Miettinen 21701	ON188808	ON188808	Finland	Viner et al. 2024
*Kneiffiellabarba-jovis*	KHL 11730	DQ873609	—	Sweden	Larsson et al. 2006
***Kneiffiellabubalina***	CLZhao 15708*	**PQ373994**	—	**China**	**Present study**
*Kneiffiellacineracea*	KAS-GEL 4958	DQ340336	—	Germany	Yurchenko et al. 2020a
*Kneiffiellacurvispora*	PRM 954540	MW345630	—	Slovakia	Běťák et al. 2021
*Kneiffiellacurvispora*	Pennanen 4040	OP620787	OP620787	Finland	Viner et al. 2024
*Kneiffielladecorticans*	SP 415980	KY081795	—	Argentina	Riebesehl and Langer 2017
*Kneiffiellaefibulata*	GB-0151167,	KY081796	—	Sweden	Riebesehl and Langer 2017
*Kneiffiellaeucalypticola*	LWZ20180515-9	MT319411	—	Australia	Luo et al. 2022
*Kneiffiellaeucalypticola*	LWZ 20180509-11*	MT319410	MT319142	China	Wang et al. 2021
*Kneiffiellafloccosa*	UC 2022902	KP814441	—	USA	Rosenthal et al. 2017
*Kneiffiellamicrospora*	Miettinen 11418	OP620788	OP620788	Indonesia	Viner et al. 2024
*Kneiffiellapalmae*	FR 7	KP689185	—	China	Wang et al. 2016
*Kneiffiellapalmae*	KAS-GEL 3456	DQ340333	—	China	Yurchenko et al. 2020a
*Kneiffiellapilaecystidiata*	MSK-F 4723	MK575208	—	Belarus	Yurchenko et al. 2020a
*Kneiffiellapilaecystidiata*	Helo 1517	OP620789	OP620789	Finland	Viner et al. 2024
*Kneiffiellapseudoabdita*	LWZ 20210624-6b*	OQ540894	—	China	Liu et al. 2024
*Kneiffiellapseudoalutacea*	LWZ 20210625-5b*	OQ540895	—	China	Liu et al. 2024
*Kneiffiellastereicola*	Blackwell 2141*	KY081797	—	USA	Riebesehl and Langer 2017
*Kneiffiellasubaltaica*	HHB-20039*	OM971679	—	USA	Langer et al. 2022
*Kneiffiellasubalutacea*	KAS-GEL 2196	DQ340341	—	Norway	Yurchenko et al. 2020a
*Kneiffiellasubalutacea*	KAS-GEL2196	DQ340341	—	Norway	Yurchenko et al. 2020a
*Kneiffiellasubefibulata*	Dai 10803	KT989971	—	China	Chen et al. 2016
*Kneiffiellasubglobosa*	Wu 890805-2	KY081798	—	Taiwan	Riebesehl and Langer 2017
*Lawrynomycescapitatus*	KHL 8464	DQ677491	DQ677491	Sweden	Cho et al. 2024
*Leifiabrevispora*	LWZ 20170820-48	MK343470	MK343474	China	Liu et al. 2019
*Leucophellinushobsonii*	Cui 6468	KT203288	KT203309	China	Cho et al. 2024
*Leucophellinusirpicoides*	Yuan 2690	KT203289	KT203310	China	Cho et al. 2024
*Lyomycesallantosporus*	FR 0249548*	KY800397	KY795963	Reunion	Yurchenko et al. 2017
*Lyomycesbambusinus*	CLZhao 4831*	MN945968	MW264919	China	Chen and Zhao 2020
*Lyomycesfimbriatus*	Wu 911204-4	MK575210	MK598740	China	Yurchenko et al. 2020a
*Lyomycesmascarensis*	KASGEL 4833*	KY800399	KY795964	Reunion	Yurchenko et al. 2020a
*Lyomycesniveus*	CLZhao 6431	MZ262541	MZ262526	China	Unpublished
*Lyomycesniveus*	CLZhao 6442	MZ262542	MZ262527	China	Unpublished
*Lyomycesochraceoalbus*	CLZhao 4385	MZ262535	MZ262521	China	Unpublished
*Lyomycesochraceoalbus*	CLZhao 4725	MZ262536	MZ262522	China	Unpublished
*Lyomycesorientalis*	LWZ 20170909-7	MT319436	MT319170	China	Luo et al. 2022
*Lyomycessambuci*	KASJR 7	KY800402	KY795966	Germany	Yurchenko et al. 2017
*Meganotuseverhartii*	JV 0108/30	MZ484529	MZ437388	USA	Unpublished
*Minimedusaobcoronata*	CBS 120605	GQ303278	GQ303309	Thailand	Cheewangkoon et al. 2009
*Minimedusapolyspora*	CBS 113.16*	MH854646	MH866167	USA	Cao et al. 2021
*Multiclavulacorynoides*	Lutzoni 930804-2	U66440	U66440	USA	Cao et al. 2021
*Multiclavulamucida*	TUB 011734	EU909345	EU909345	Germany	Cao et al. 2021
*Multiclavulapetricola*	356 ex*	LC516464	LC516465	Japan	Cao et al. 2021
*Multiclavulavernalis*	Lutzoni 930806-1	U66439	U66439	USA	Cao et al. 2021
*Muscinuptalaevis*	V. Haikonen 19745	MF319066	MF318921	Finland	Cho et al. 2024
*Neoburgoafreyi*	LF1256*	KX423756	KX423756	Switzerland	Lawrey et al. 2016
*Neoburgoafreyi*	JL596-16	KX423754	KX423755	Switzerland	Lawrey et al. 2016
*Neomensulariaduplicata*	LWZ 20150529-4	KX078217	KX078221	China	Wu et al. 2016
*Neophellinusuncisetus*	MUCL 47061	GU461972	GU462000	Argentina	Amalfi et al. 2010
*Nigrohirschioporusdurus*	Dai 20642	OL470321	OL462835	China	Cho et al. 2024
*Nigrohirschioporusdurus*	He 20120724-11	OQ448973	OQ449076	China	Cho et al. 2024
*Nigrohirschioporusgriseofuscus*	B3942	OQ448975	OQ438022	Brazil	Cho et al. 2024
*Nigrohirschioporusgriseofuscus*	JV 1909/ 6	OQ437343	OQ438024	French Guiana	Cho et al. 2024
*Nigrohirschioporussector*	AS 2707	OQ437344	OQ438025	Brazil	Cho et al. 2024
*Nigrohirschioporustrimiticus*	B696*	OQ453308	OQ453535	Brazil	Cho et al. 2024
*Nothophellinusandinopatagonicus*	JV 1911/20	MZ484532	MZ437391	Chile	Wu et al. 2022
*Oliveoniasubfibrillosa*	TH 2018074	MT235650	MT235618	Finland	Cao et al. 2021
*Oliveoniasubfibrillosa*	TH 2018179	MT235649	MT235617	Finland	Cao et al. 2021
*Oliveoniasubfibrillosa*	VS 9048	MT235647	MT235615	Russia	Cao et al. 2021
*Oliveoniasubfibrillosa*	VS 9053	MT235645	MT235614	Russia	Cao et al. 2021
*Onniatomentosa*	Dai 22935	OL473604	OL473617	China	Cho et al. 2024
*Onniatomentosa*	Niemela 9079	MF319075	MF318931	Finland	Wu et al. 2022
*Pachynotuspunctatus*	Dai 17803*	MZ484535	MZ437394	Singapore	Wu et al. 2022
*Pallidohirschioporusbiformis*	Dai 12746	OQ453311	OQ453538	USA	Cho et al. 2024
*Pallidohirschioporusbiformis*	Dai 19466	OQ453223	OQ453548	China	Cho et al. 2024
*Pallidohirschioporusbrastagii*	Dai 22919	OQ453371	OQ453297	China	Cho et al. 2024
*Pallidohirschioporuspolycystidiatus*	Dai 19100	OQ453378	OQ453301	China	Cho et al. 2024
*Pallidohirschioporusversicolor*	Dai 19331	OQ453386	OQ474951	China	Cho et al. 2024
*Peniophorellaaspersa*	CLZhao 17063	OM985730	OM985771	China	Cho et al. 2024
*Peniophorellaaspersa*	F24809*	NR_172775	NG_073750	China	Yurchenko et al. 2020b
*Peniophorellacrystallifera*	F23666*	NR_171802	NG_073751	China	Yurchenko et al. 2020b
*Peniophorellacrystallifera*	LWZ 20210626-4a	ON063685	ON063885	China	Wang et al. 2023
*Peniophorellafissurata*	CLZhao 9421*	NR177497	NG154027	China	Guan et al. 2020
*Peniophorellaodontiiformis*	CLZhao 9862	MT247004	OM985779	China	Cho et al. 2024
*Peniophorellaodontiiformis*	SFC20150108-37	OQ996168	OQ996199	South Korea	Cho et al. 2024
*Peniophorellapallida*	CLZhao 3017	OM985738	OM985780	China	Cho et al. 2024
*Peniophorellapraetermissa*	AFTOL-ID 518	AY854081	AY700185	USA	Cho et al. 2024
*Peniophorellapraetermissa*	LWZ 20180903-14	ON063686	ON063886	China	Wang et al. 2023
*Peniophorellapubera*	LWZ 20210624-16b	ON063687	ON063887	China	Wang et al. 2023
*Peniophorellareticulata*	F22559*	NR172776	NG073752	China	Yurchenko et al. 2020b
*Peniophorellarude*	F30649	MN062105	MN062153	China	Yurchenko et al. 2020b
*Peniophorellarude*	LWZ 20171026-7	ON063688	ON063888	China	Wang et al. 2023
*Peniophorellasubpraetermissa*	LWZ 20190816-3b	ON063689	ON063889	China	Wang et al. 2023
*Perennihirschioporusvariabilis*	B856	OQ474942	OQ474957	Brazil	Cho et al. 2024
*Perenninotusshoreicola*	Dai 13614	KJ575522	KT749416	Thailand	Wu et al. 2022
*Phellinidiumferrugineofuscum*	Cui 10042	KC782527	KC782529	China	Wu et al. 2022
*Phellinopsisconchata*	L-7601	KU139188	KU139257	USA	Brazee 2015
*Phylloporiagabonensis*	MUCL 55571	NR_154331	NG_059641	Gabon	Unpublished
*Phylloporianodostipitata*	FLOR 51173*	KJ639055	KJ631412	Brazil	Wu et al. 2022
*Phylloporiaperangusta*	Dai 18139	MH151169	MG738803	China	Wu et al. 2022
*Porodaedaleapini*	No-6170-T	JX110037	JX110081	Porrugal	Brazee and Lindner 2013
*Pseudoinonotusdryadeus*	JV 1907/7	MZ484540	MZ437400	Czechia	Wu et al. 2022
*Pyrrhodermaadamantinum*	Dai 13832	MF860790	MF860736	China	Wu et al. 2022
*Resiniciumaustroasianum*	LWZ 20180417-5*	NR173962	NG088188	China	Yu et al. 2021
*Resiniciumaustroasianum*	LWZ 20191208-11	ON063691	ON063891	China	Wang et al. 2023
*Resiniciumbicolor*	AFTOL-810	DQ218310	AF393061	USA	Cho et al. 2024
*Resiniciumlateastrocystidium*	LWZ 20180414-15*	NR173963	MW414455	China	Yu et al. 2021
*Resiniciumlateastrocystidium*	LWZ 20180416-10	MW414510	MW414456	China	Yu et al. 2021
*Resiniciummonticola*	FP-150360*	NR_111226	DQ863697	Jamaica	Cho et al. 2024
*Resiniciummutabile*	FP-102989*	NR_119612	DQ863699	Puerto Rico	Cho et al. 2024
*Resiniciumrimulosum*	KUC20131022-12	KJ668464	KJ668315	South Korea	Jang et al. 2016
*Rhizoctoniasolani*	BRS17	MK481078	MN078809	India	Cao et al. 2021
*Rickenellafibula*	HBK013	MF319081	MF318941	USA	Cho et al. 2024
*Rickenellafibula*	SFC20230704-06	OR758634	OR758646	South Korea	Cho et al. 2024
*Rickenellafibula*	RAS051	MF319094	MF318972	USA	Cho et al. 2024
*Rickenellafibula*	HBK016	MF319084	MF318944	USA	Cho et al. 2024
*Rickenellafibula*	HBK014	MF319082	MF318942	USA	Cho et al. 2024
*Rickenellaindica*	SFC20140626-39	OQ996172	OQ996203	South Korea	Cho et al. 2024
*Rickenellamellea*	CBS 579.87	MH862106	MH873795	France	Vu et al. 2018
*Rickenellamellea*	CBS 581.87	MH862107	MH873796	France	Vu et al. 2018
*Rickenellaminuta*	MES1950	MF319097	MF318964	Argentina	Cho et al. 2024
*Rickenellaminuta*	MES1892	MF318966	MF288881	Argentina	Cho et al. 2024
*Rickenellaminuta*	MES1965	MF319105	MF318963	Argentina	Cho et al. 2024
*Rickenellaumbelliformis*	SFC20150701-65*	OQ996173	OQ996204	South Korea	Cho et al. 2024
*Rickenellaumbelliformis*	SFC20160713-77	OQ996175	OQ996205	South Korea	Cho et al. 2024
*Rickenellaumbelliformis*	SFC20180704-81	OQ996176	OQ996206	South Korea	Cho et al. 2024
*Rigidoporuscorticola*	KUC20130718-79	KJ668502	KJ668354	South Korea	Jang et al. 2016
*Rigidoporuscorticola*	LWZ 20190819-3b	ON063673	ON063872	China	Wang et al. 2023
*Rigidoporuscorticola*	SFC20230816-48	OR758635	OR758647	South Korea	Cho et al. 2024
*Rigidoporuscuneatus*	Cui 10855	OQ930254	OQ924530	South Korea	Cho et al. 2024
*Rigidoporuscuneatus*	Dai 7339	KT203294	KT203315	China	Cho et al. 2024
*Rigidoporusginkgonis*	Cui 5555	KT203295	KT203316	China	Cho et al. 2024
*Rigidoporusginkgonis*	SFC20230630-23	OR758636	OR758648	South Korea	Cho et al. 2024
*Rigidoporusjuniperinus*	YG 1070	MK433641	MK433643	Uzbekistan	Cho et al. 2024
*Rigidoporusjuniperinus*	Dai 17100	OQ930261	OQ924537	Uzbekistan	Cho et al. 2024
*Rogersiomycesmalaysianus*	LE-BIN 3507-10	KT779285	KU820986	Vietnam	Cao et al. 2021
*Sanghuangporusweigelae*	LWZ 20210623-2a	ON063671	ON063870	China	Wang et al. 2023
*Sanghuangporuszonatus*	Dai 10841	OP962417	KP030775	China	Wu et al. 2022
*Schizocorticiumlenis*	LWZ 20180921-7*	MW414521	MW414467	China	Yu et al. 2021
*Schizocorticiummagnosporum*	Wu 1510-34*	MK405351	MK405337	China	Wang and Zhou 2024
*Schizocorticiummediosporum*	Chen 2456*	MK405359	MK405345	China	Wang and Zhou 2024
*Schizocorticiumparvosporum*	GC 1508-127*	MK405361	MK405347	China	Wang and Zhou 2024
*Sideraminutipora*	Cui 16720	MN621349	MN621348	Australia	Wang and Zhou 2024
*Sideraminutissima*	Dai 19529	MN621352	MN621350	Sri Lanka	Du et al. 2020
*Sideraparallela*	Dai 22038	MW477793	MW474964	China	Cho et al. 2024
*Siderasrilankensis*	Dai 19654*	NR_172780	NG_075310	Sri Lanka	Cho et al. 2024
*Sideratenuis*	Dai 18697*	NR_171833	NG_075283	Singapore	Cho et al. 2024
*Sideratibetica*	SFC20230317-17	OQ996177	OQ996207	South Korea	Cho et al. 2024
*Sideratibetica*	Dai 23648*	NR_177641	OM974245	China	Liu et al. 2022
*Sideravesiculosa*	BJFC 025377*	NR164588	NG066418	Singapore	Cho et al. 2024
*Sideravulgaris*	Dai 21057	MW198484	MW192009	Singapore	Liu et al. 2021
*Sistotremaconﬂuens*	FCUG298	DQ267125	AY647214	USA	Cao et al. 2021
*Sistotremaconﬂuens*	PV174	AY463466	AY586712	Czechia	Larsson et al. 2004
*Sistotremasubconfluens*	Dai 12577	JX076812	JX076810	China	Zhou and Qin 2013
*Sistotremellaperpusilla*	CBS 126048	MH864061	MH875516	USA	Cao et al. 2021
*Skvortzoviadabieshanensis*	LWZ 20201012-22*	NR_173964	NG_088189	China	Yu et al. 2021
*Skvortzoviadabieshanensis*	LWZ 20210918-15b	ON063694	ON063894	China	Yu et al. 2021
*Skvortzoviapinicola*	LWZ 20210623-18b	ON063695	ON063895	China	Wang et al. 2023
*Skvortzoviaqilianensis*	LWZ 20180904-16*	NR173965	NG088190	China	Yu et al. 2021
*Skvortzoviaqilianensis*	LWZ 20180904–20	MW414520	MW414466	China	Yu et al. 2021
*Skvortzoviayunnanensis*	CLZhao 16084	MW472754	MW473473	China	Dong et al. 2021
*Trichaptumbyssogenum*	Dai 15555	OQ449085	OQ449026	China	Cho et al. 2024
*Trichaptumperrottetii*	JV 1908/ 45	OQ449092	OQ449031	French Guiana	Cho et al. 2024
*Trichosporoninsectorum*	CBS 10422*	KF036603	KY109953	Panama	Cao et al. 2021
*Tubulicrinisaccedens*	MICH:352299	OL756001	OL742444	USA	Cho et al. 2024
*Tubulicriniscalothrix*	LWZ 20210919-1b	ON063704	ON063904	China	Wang et al. 2023
*Tubulicrinisglebulosus*	LWZ 20180903-13	ON063705	ON063905	China	Wang et al. 2023
*Tubulicrinissubulatus*	LWZ 20190914-7	ON063706	ON063906	China	Wang et al. 2023
*Tubulicrinisxantha*	CLZhao 2869	MT153875	MT153882	China	He et al. 2020
*Tulasnellaasymmetrica*	AFTOL-ID 1678	DQ520101	DQ520101	Germany	Cao et al. 2021
*Tulasnellairregularis*	CBS 574.83	NR160166	NG057720	Australia	Cao et al. 2021
*Tulasnellapruinosa*	DAOM 17641	DQ457642	AF518662	USA	Cao et al. 2021
*Tulasnellaviolea*	AFTOL-ID 1879	DQ520097	DQ520097	Germany	Cao et al. 2021
*Xylodonacuminatus*	Larsson 16029	ON197552	—	Brazil	Viner et al. 2023
*Xylodonacystidiatus*	LWZ 20180514-9*	MT319474	MT319211	Australia	Luo et al. 2022
*Xylodonafromontanus*	H 7006811*	OQ645463	—	Rwanda	Yurchenko et al. 2024
*Xylodonangustisporus*	Ryvarden 50691b*	OK273831	—	Cameroon	Viner et al. 2021
*Xylodonapacheriensis*	Wu 0910-58	KX857797	KX857822	China	Chen et al. 2017
*Xylodonapacheriensis*	Canfield 180	KY081800	—	USA	Wang et al. 2021
*Xylodonasiaticus*	CLZhao 2282	OM959481	OM967416	China	Zhang et al. 2024a
*Xylodonasiaticus*	CLZhao 10368*	OM959479	OM967417	China	Zhang et al. 2024a
*Xylodonasiaticus*	CLZhao 10430	OM959480	OM967418	China	Zhang et al. 2024a
*Xylodonasiaticus*	CLZhao 2282	OM959481	OM967416	China	Zhang et al. 2024a
*Xylodonasperus*	Spirin 11923	OK273838	—	Russia	Viner et al. 2021
*Xylodonastrocystidiatus*	TNM F24764	NR154054	—	China	Yurchenko and Wu 2014
*Xylodonastrocystidiatus*	Wu 9211-71*	JN129972	JN129973	China	Yurchenko and Wu 2014
*Xylodonattenuatus*	Spirin 8775*	MH324476	—	America	Wang et al. 2021
*Xylodonaustralis*	LWZ 20180509-8	MT319503	—	China	Wang et al. 2021
*Xylodonbambusinus*	CLZhao 11310*	MW394660	—	China	Ma and Zhao 2021
*Xylodonbambusinus*	CLZhao 9174	MW394657	MW394650	China	Ma and Zhao 2021
*Xylodonbambusinus*	CLZhao 9174	MW394657	MW394650	China	Ma and Zhao 2021
*Xylodonborealis*	JS 26064	AY463429	—	Norway	Larsson et al. 2004
*Xylodonbrevisetus*	JS 17863	AY463428	—	Norway	Larsson et al. 2004
*Xylodoncrystalliger*	LWZ 20170816-33	MT319521	MT319269	China	Luo et al. 2022
*Xylodoncrystalliger*	KUN 2312*	NR166242	—	China	Viner et al. 2018
*Xylodoncymosus*	Miettinen 19606*	ON197554	—	USA	Viner et al. 2023
*Xylodoncystidiatus*	FR 0249200	MH880195	MH884896	France	Riebesehl et al. 2019
*Xylodondamansaraensis*	LWZ 20180417-23	MT319499	—	Malaysia	Wang et al. 2021
*Xylodondaweishanense*	CLZhao 18357	OP730715	—	China	Guan et al. 2023
*Xylodondaweishanense*	CLZhao 18492	OP730719	—	China	Guan et al. 2023
*Xylodondetriticus*	Zíbarová 30.10.17	MH320793	—	Czech Republic	Wang et al. 2021
*Xylodondissiliens*	Ryvarden 44817*	OK273856	—	Uganda	Viner et al. 2021
*Xylodonechinatus*	OM 18237	OQ645464	—	Indonesia	Yurchenko et al. 2024
*Xylodonexilis*	TUB-FO42565*	MH880198	MH884898	China	Riebesehl et al. 2019
*Xylodonfilicinus*	MSKF 12869*	MH880199	NG067836	China	Riebesehl et al. 2019
*Xylodonfissuratus*	CLZhao 9407*	OP730714	—	China	Guan et al. 2023
*Xylodonflaviporus*	FR-0249797	MH880201	MH884901	Reunion	Riebesehl et al. 2019
*Xylodonflocculosus*	CLZhao 18342*	MW980776	—	China	Qu and Zhao 2022
*Xylodonfollis*	FR-0249814*	MH880204	MH884902	Reunion	Riebesehl et al. 2019
*Xylodongloeocystidiifer*	BLS M-5232*	OQ645467	—	Ecuador	Yurchenko et al. 2024
*Xylodongloeocystidiifer*	EYu 190720-11	OR240822	—	Ecuador	Unpublished
*Xylodongossypinus*	CLZhao 4465	MZ663803	MZ663812	China	Luo et al. 2021
*Xylodongossypinus*	CLZhao 8375*	MZ663804	—	China	Luo et al. 2021
*Xylodongossypinus*	CLZhao 4465	MZ663803	MZ663812	China	Luo et al. 2021
*Xylodongrandineus*	CLZhao 6425	OM338090	—	China	Luo et al. 2022
***Xylodongranulanoides***	CLZhao 17253*	**PQ373995**	**PQ373981**	**China**	**Present study**
***Xylodongranulans***	**CLZhao 17804**	**PQ373996**	—	**China**	**Present study**
***Xylodongranulans***	CLZhao 17866*	**PQ373997**	**PQ373982**	**China**	**Present study**
*Xylodonhastifer*	K(M) 172400*	NR166558	—	America	Riebesehl and Langer 2017
*Xylodonheterocystidiatus*	LWZ20180921-19	MT319676	MT319266	Australia	Zhang et al 2024a
*Xylodonheterocystidiatus*	Wei 17-314	MT731753	MT731754	China	Zhang et al. 2024a
*Xylodonhjortstamii*	Gorjon 3187	ON188816	—	Chile	Yuan and Zhao 2024
*Xylodonhyphodontinus*	KAS-GEL9222	MH880205	—	Kenya	Riebesehl et al. 2019
*Xylodonjacobaeus*	MA-Fungi 91340*	MH430073	—	Spain	Wang et al. 2021
*Xylodonkunmingensis*	TUB-FO 42565	MH880198	—	China	Wang et al. 2021
*Xylodonlaceratus*	CLZhao 9892*	OL619258	OL619266	China	Qu et al. 2022
*Xylodonlagenicystidiatus*	LWZ 20180513-16*	MT319634	MT319368	Australia	Luo et al. 2022
*Xylodonlanatus*	CFMR FP-101864-A*	OQ645474	—	USA	Yurchenko et al. 2024
*Xylodonlenis*	Wu 890714-3	KY081802	—	China	Riebesehl et al. 2019
*Xylodonmacrosporus*	CLZhao 10226*	MZ663809	MZ663817	China	Luo et al. 2021
*Xylodonmagallanesii*	MA: Fungi:90397*	MT158729	—	Chile	Fernandez-Lopez et al. 2020
*Xylodonmantiqueirensis*	MV 529	OQ645478	—	Brazil	Yurchenko et al. 2024
*Xylodonmollissimus*	LWZ 20160318-3*	KY007517	MT319347	China	Luo et al. 2022
*Xylodonmontanus*	CLZhao 8179*	OL619260	OL619268	China	Qu et al. 2022
*Xylodonmuchuanensis*	LWZ 20200819-3a	OQ540903	—	China	Unpublished
*Xylodonmuchuanensis*	LWZ 20200819-2b*	OQ540902	—	China	Unpublished
*Xylodonneotropicus*	MV 580	OQ645479	—	Brazil	Yurchenko et al. 2024
*Xylodonnesporii*	LWZ 20180921-35	MT319655	MT319238	China	Luo et al. 2022
*Xylodonniemelaei*	LWZ 20150707-13	MT319630	MT319365	China	Luo et al. 2022
*Xylodonnongravis*	GC 1412-22*	KX857801	KX857818	China	Chen et al. 2017
*Xylodonnothofagi*	ICMP 13842	AF145583	—	China	Wang et al. 2021
*Xylodonovisporus*	LWZ 20170815-31	MT319666	MT319346	China	Luo et al. 2022
*Xylodonpapillosus*	CBS 114.71	MH860026	—	Netherlands	Vu et al. 2018
*Xylodonparadoxus*	Dai 14983	MT319519	MT319267	China	Luo et al. 2022
*Xylodonpatagonicus*	ICMP 13832	AF145581	—	Argentina	Wang et al. 2021
*Xylodonpruinosus*	Spirin 2877	MH332700	—	Estonia	Wang et al. 2021
*Xylodonpruniaceus*	Ryvarden 11251	OK273828	—	Malawi	Viner et al. 2021
*Xylodonpseudolanatus*	FP 150922*	MH880220	NG067837	Belize	Riebesehl et al. 2019
*Xylodonpseudotropicus*	Dai 16167	MT319509	MT319255	China	Luo et al. 2022
*Xylodonpseudotropicus*	Dai 10768*	KF917543	—	China	Wang et al. 2021
*Xylodonpseudotropicus*	Dai 16167	MT319509	MT319255	China	Luo et al. 2022
*Xylodonpuerensis*	CLZhao 8142*	OP730720	—	China	Guan et al. 2023
*Xylodonpunctus*	CLZhao 17691*	OM338092	—	China	Luo et al. 2022
*Xylodonpunctus*	CLZhao 17908	OM338093	—	China	Luo et al. 2022
*Xylodonquercinus*	Otto Miettinen 15050,1	KT361632	—	Finland	Ariyawansa et al. 2015
*Xylodonquercinus*	Spirin 12030	OK273841	—	Russia	Viner et al. 2021
*Xylodonquercinus*	Otto Miettinen 15050,1	KT361632	—	Finland	Ariyawansa et al. 2015
*Xylodonquercinus*	KHL 11076	KT361633	AY586678	Sweden	Larsson et al. 2004
*Xylodonquercinus*	KHL 11076	KT361633	AY586678	Sweden	Larsson et al. 2004
*Xylodonraduloides*	FCUG 2433	AF145570	—	Russia	Wang et al. 2021
*Xylodonramicida*	Spirin 7664*	NR138013	—	America	Yuan and Zhao 2024
*Xylodonreticulatus*	Wu 1109-178	KX857805	—	China	Wang et al. 2021
*Xylodonreticulatus*	GC 1512-1	KX857808	—	China	Wang et al. 2021
*Xylodonrhizomorphus*	Dai 12367*	NR154067	—	China	Wang et al. 2021
*Xylodonrhododendricola*	LWZ 20180513-9	MT319621	MT319357	Australia	Luo et al. 2022
*Xylodonrimosissimus*	Ryberg 021031	DQ873627	DQ873628	Sweden	Larsson et al. 2006
*Xylodonserpentiformis*	LWZ 20170816-15	MT319673	MT319218	China	Luo et al. 2022
*Xylodonsinensis*	CLZhao 9197	MZ663810	MZ663818	China	Luo et al. 2021
*Xylodonsinensis*	CLZhao 11120*	OK560885	—	China	Luo et al. 2021
*Xylodonspathulatus*	LWZ 20180804-10	MT319646	MT319354	China	Luo et al. 2022
*Xylodonsubclavatus*	FO 42167	MH880232	—	China	Wang et al. 2021
*Xylodonsubflaviporus*	Wu 0809-76	KX857803	KX857815	China	Chen et al. 2017
*Xylodonsubflaviporus*	TNM F29958*	NR184880	—	China	Chen et al. 2017
*Xylodonsubpunctus*	CLZhao 6165	PP537958	—	China	Unpublished
*Xylodonsubpunctus*	CLZhao 31242*	PP537957	—	China	Unpublished
*Xylodonsubserpentiformis*	LWZ 20180512-16	MT319486	MT319226	Australia	Luo et al. 2022
*Xylodonsubtilissimus*	Spirin 12228	ON188818	—	Russia	Yuan and Zhao 2024
*Xylodonsubtropicus*	LWZ 20180510-24	MT319541	MT319308	China	Luo et al. 2022
*Xylodontaiwanianus*	CBS 125875	MH864080	MH875537	New Zealand	Vu et al. 2018
*Xylodontropicus*	CLZhao 3351*	OL619261	OL619269	China	Qu et al. 2022
*Xylodonussuriensis*	KUN 1989*	NR166241	—	America	Yuan and Zhao 2024
*Xylodonverecundus*	KHL 12261	DQ873642	DQ873643	Sweden	Larsson et al. 2006
*Xylodonvictoriensis*	LWZ 20180510-29	MT319487	MT319228	Australia	Luo et al. 2022
*Xylodonwenshanensis*	CLZhao 15729*	OM338097	—	China	Luo et al. 2022
*Xylodonwenshanensis*	CLZhao 10790	OM338095	—	China	Luo et al. 2022
*Xylodonwenshanensis*	CLZhao 15782	OM338098	—	China	Luo et al. 2022
*Xylodonwenshanensis*	CLZhao 15718	OM338096	—	China	Luo et al. 2022
*Xylodonxinpingensis*	CLZhao 11224	MW394662	MW394654	China	Ma and Zhao 2021
*Xylodonxinpingensis*	CLZhao 9174*	MW394657	—	China	Ma and Zhao 2021
*Xylodonyarraensis*	LWZ 20180510-5	MT319639	MT319378	Australia	Luo et al. 2022
*Xylodonyunnanensis*	LWZ 20180922-47	MT319660	—	China	Wang et al. 2021

### ﻿Phylogenetic analyses

Sequences generated for this study were aligned with additional sequences downloaded from GenBank. Sequences were aligned using MAFFT v.7 (https://mafft.cbrc.jp/alignment/server/), adjusting the direction of nucleotide sequences according to the first sequence (accurate enough for most cases), and selecting the G-INS-i iterative refinement method (Katoh et al. 2019). Alignments were manually adjusted to maximize alignment and minimize gaps with BioEdit v.7.0.9 (Hall 1999). A dataset of concatenated ITS and nLSU sequences was used to determine the phylogenetic position of the new species. Maximum likelihood (ML) analysis was performed using the CIPRES Science Gateway based on the dataset using the RA×ML-HPC BlackBox tool, with setting RA×ML halt bootstrapping automatically and 0.25 for maximum hours and obtaining the best tree using ML search (Miller et al. 2010). Other parameters in ML analysis used default settings, and statistical support values were obtained using nonparametric bootstrapping with 1,000 replicates. Bayesian inference (BI) analysis based on the dataset was performed using MrBayes v.3.2.6 (Ronquist and Huelsenbeck 2003). The best substitution model for the dataset was selected by ModelFinder using a Bayesian information criterion, and the model was used for Bayesian analysis (Kalyaanamoorthy et al. 2017). Four Markov chains were run from random starting trees. Trees were sampled every 1.00^th^ generation. The first 25% of sampled trees were discarded as burn-in, whereas other trees were used to construct a 50% majority consensus tree and for calculating Bayesian posterior probabilities (BPPs).

The branches of the consensus tree that received bootstrap support for ML were greater than or equal to 70%, and Bayesian posterior probabilities were greater than or equal to 0.95, respectively.

## ﻿Results

**Figure 1.**
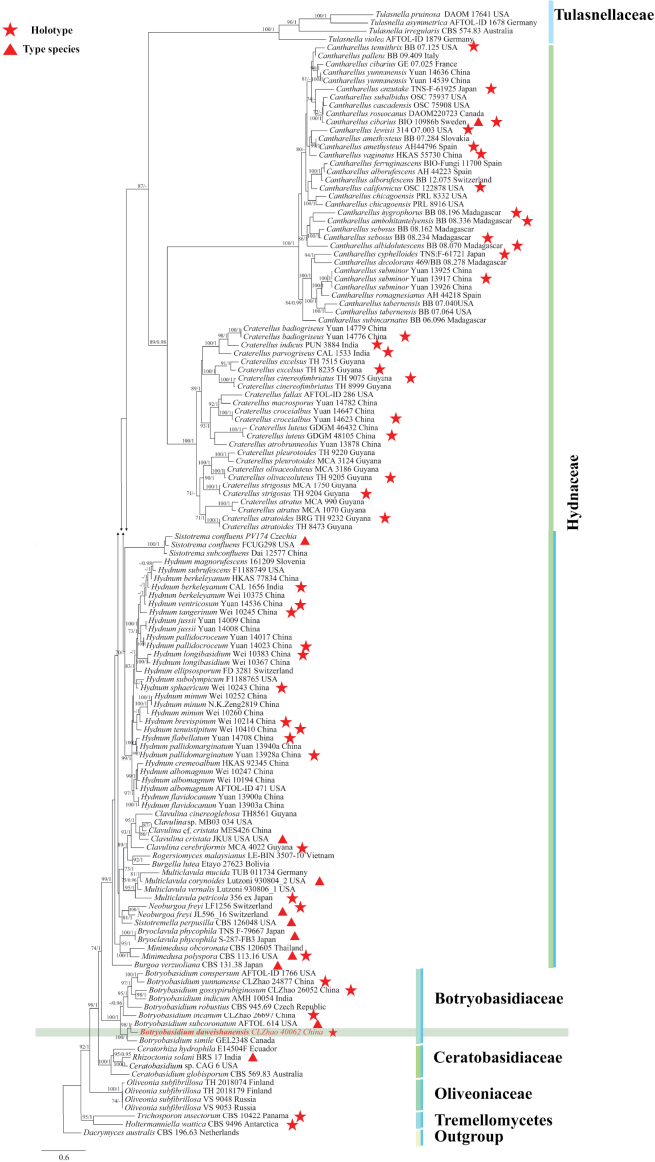
Phylogeny of species in Cantharellales generated by maximum likelihood based on ITS+nLSU sequence data. Branches are labeled with maximum likelihood bootstrap ≥ 70% and Bayesian posterior probabilities ≥ 0.95, respectively.

**Figure 2.**
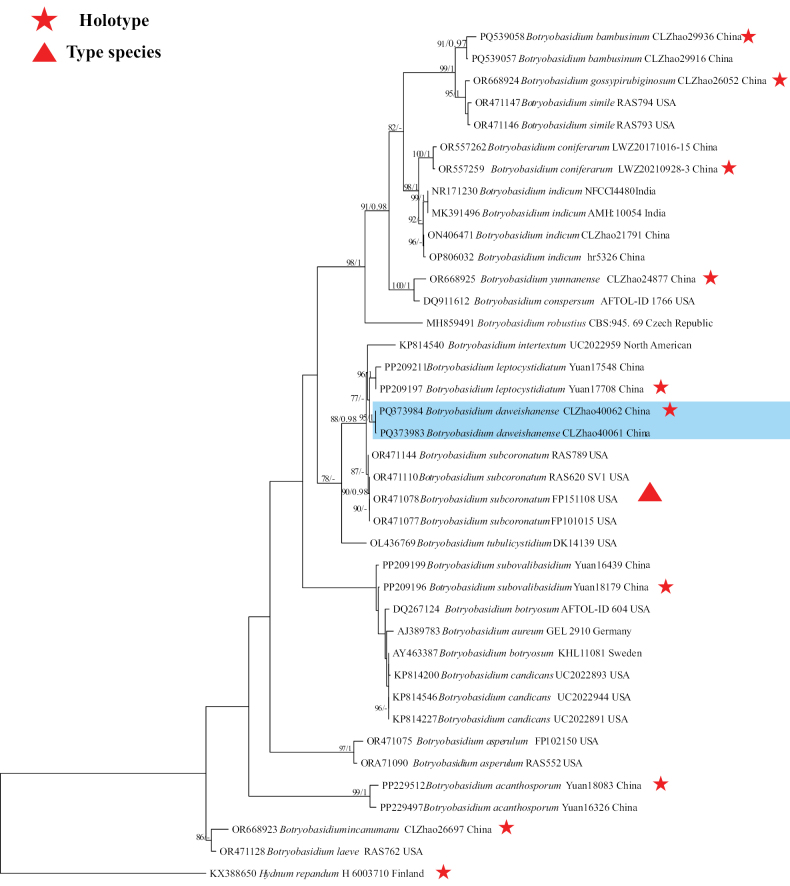
Phylogeny of species in *Botryobasidium* generated by maximum likelihood based on ITS sequence data. Branches are labeled with maximum likelihood bootstrap ≥ 70% and Bayesian posterior probabilities ≥ 0.95, respectively.

**Figure 3.**
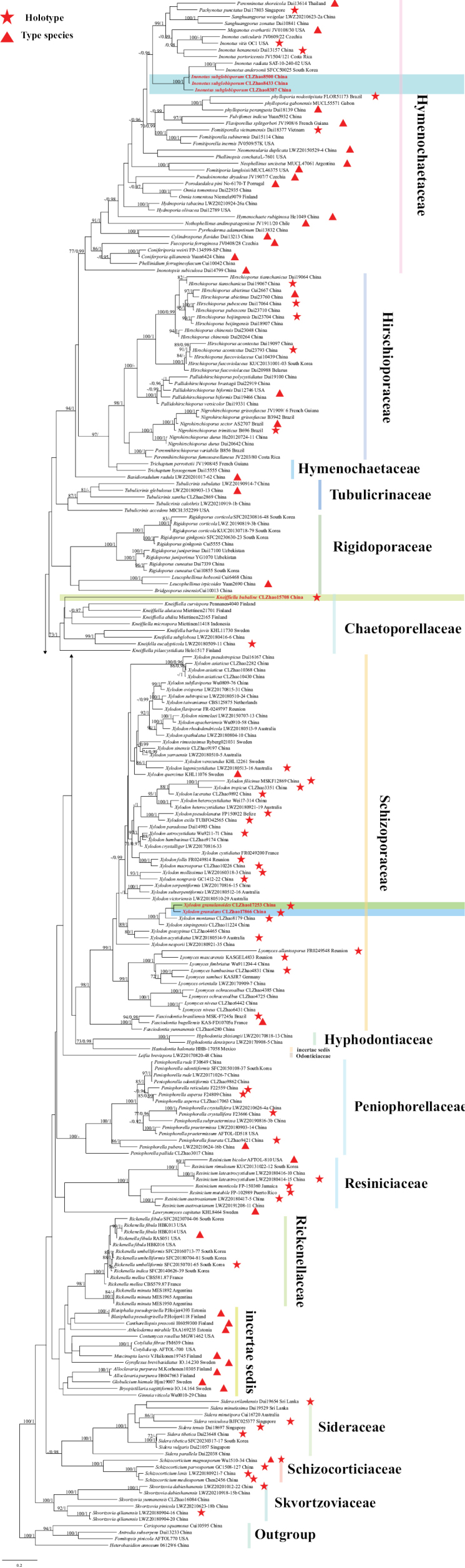
Phylogeny of species in Hymenochaetales generated by maximum likelihood based on ITS+nLSU sequence data. Branches are labeled with maximum likelihood bootstrap ≥ 70% and Bayesian posterior probabilities ≥ 0.95, respectively.

**Figure 4.**
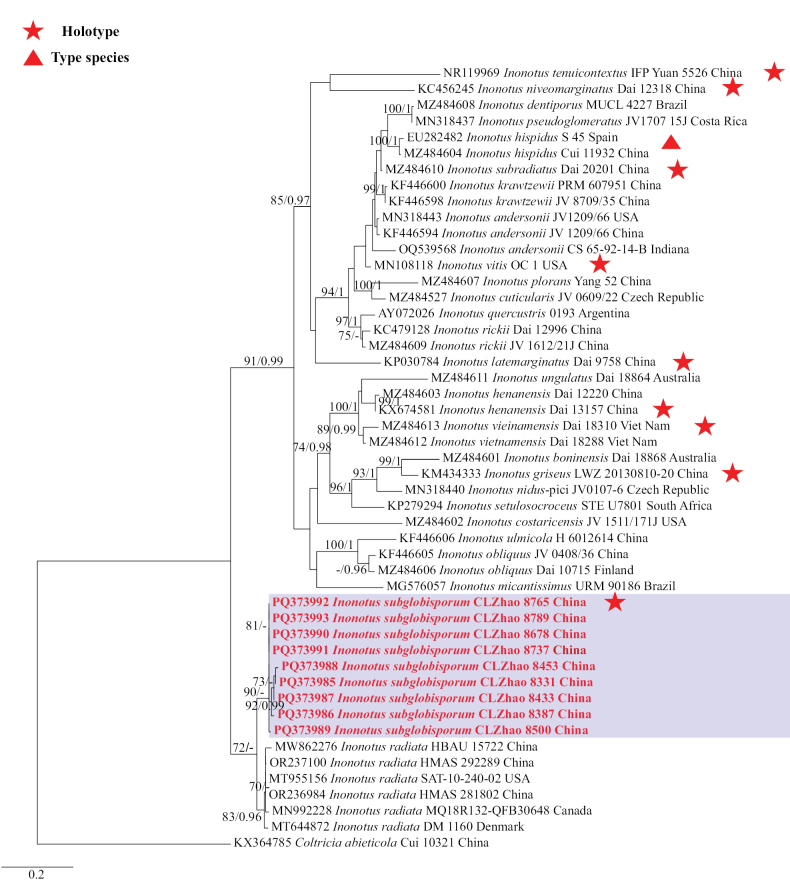
Phylogeny of species in *Inonotus* generated by maximum likelihood based on ITS sequence data. Branches are labeled with maximum likelihood bootstrap ≥ 70% and Bayesian posterior probabilities ≥ 0.95, respectively.

**Figure 5.**
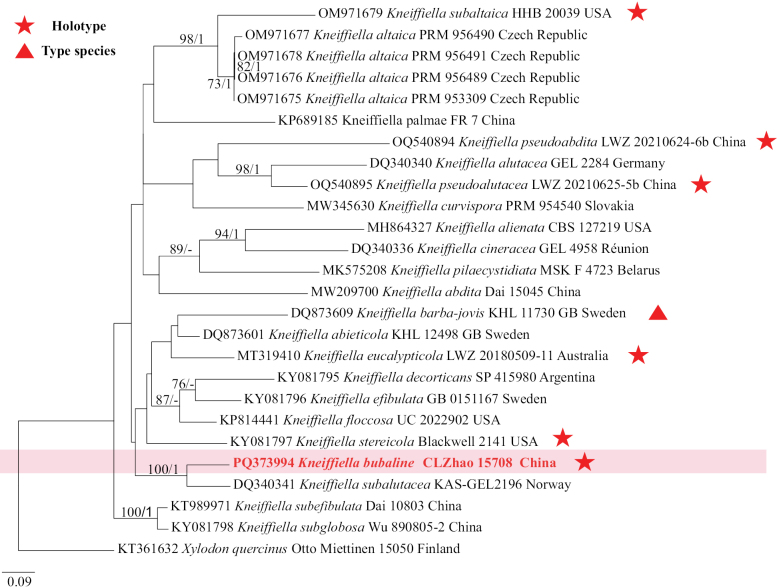
Phylogeny of species in *Kneiffiella* generated by maximum likelihood based on ITS sequence data. Branches are labeled with maximum likelihood bootstrap ≥ 70% and Bayesian posterior probabilities ≥ 0.95, respectively.

**Figure 6.**
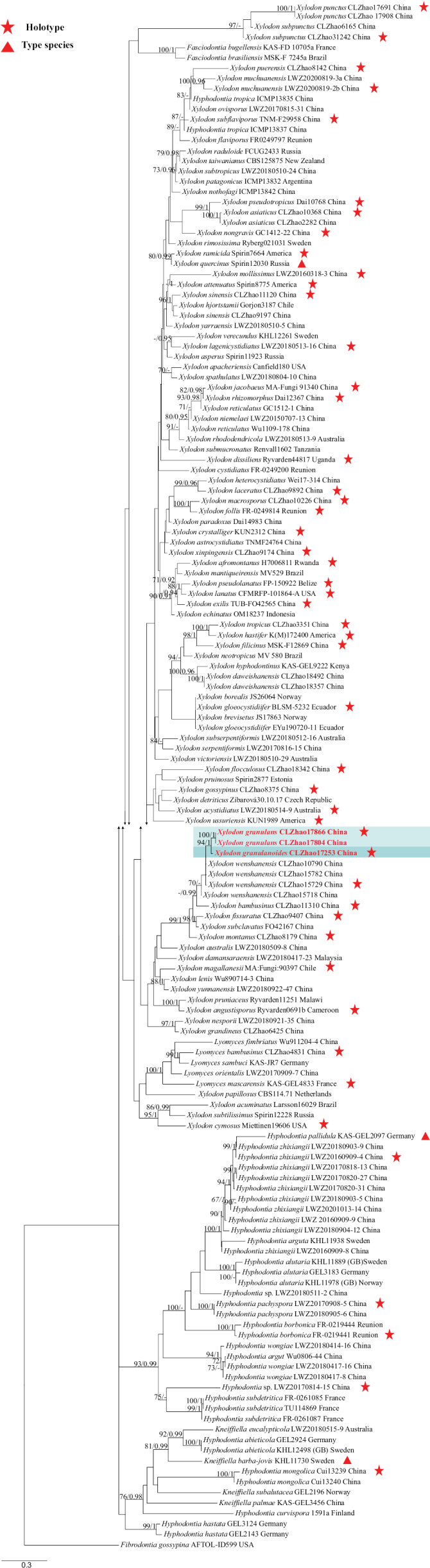
Phylogeny of species in *Xylodon* generated by maximum likelihood based on ITS sequence data. Branches are labeled with maximum likelihood bootstrap ≥ 70% and Bayesian posterior probabilities ≥ 0.95, respectively.

BI analysis yielded a similar topology to MP and ML analysis; thus, the MP tree is provided (Figs 1–6). Branches that received bootstrap support for ML (ML-BS) and BI (BPP) greater than or equal to 70% (MP-BS and ML-BS) and 0.95 (BPP) were considered as significantly supported, respectively.

### ﻿The phylogeny of Cantharellales

The ITS and nLSU dataset contained sequences from 135 fungal specimens representing 97 Cantharellales taxa. The average SD of split frequencies in BI analyses is 0.013339 (BI), and the effective sample size (ESS) for Bayesian analysis across the two runs is double the average ESS (avg ESS) = 725. The phylogenetic tree (Fig. 1) reveals that the Cantharellales new species were grouped into the genus *Botryobasidium* (Botryobasidiaceae).

### ﻿The phylogeny of *Botryobasidium*

The ITS dataset contained sequences from 39 fungal specimens representing 22 *Botryobasidium* taxa. The average SD of split frequencies in BI analyses is 0.006652 (BI), and the effective sample size (ESS) for Bayesian analysis across the two runs is double the average ESS (avg ESS) = 1700.5. The phylogenetic tree (Fig. 2) reveals that the new species groups with three taxa, *Botryobasidiumintertextum* (Schwein.) Jülich & Stalper, *B.leptocystidiatum* L.J. Zhou & H.S. Yuan, and *B.subcoronatum*.

### ﻿The phylogeny of Hymenochaetales

The ITS dataset contained sequences from 239 fungal specimens representing 196 Hymenochaetales taxa. The average SD of split frequencies in BI analyses is 0.013469 (BI), and the effective sample size (ESS) for Bayesian analysis across the two runs is double the average ESS (avg ESS) = 671.5. The phylogenetic tree (Fig. 3) reveals the four new Hymenochaetales species grouped into Hymenochaetaceae, Hyphodontiaceae, and Schizoporaceae.

### ﻿The phylogeny of *Inonotus*

The ITS dataset contained sequences from 49 fungal specimens representing 26 *Inonotus* taxa. The average SD of split frequencies in BI analyses is 0.004227 (BI), and the effective sample size (ESS) for Bayesian analysis across the two runs is double the average ESS (avg ESS) = 2018.5. The phylogenetic tree (Fig. 4) reveals that the new species is closely related to *Inonotusradiatus* (Sowerby) P. Karst.

### ﻿The phylogeny of *Kneiffiella*

The ITS dataset contained sequences from 26 fungal specimens representing 21 *Kneiffiella* taxa. The average SD of split frequencies in BI analyses is 0.007515 (BI), and the effective sample size (ESS) for Bayesian analysis across the two runs is double the average ESS (avg ESS) = 4107.5. The phylogenetic tree (Fig. 5) reveals that the new species is closely related to *Kneiffiellasubalutacea* (P. Karst.) Jülich & Stalpers.

### ﻿The phylogeny of *Xylodon*

The ITS dataset contained sequences from 149 fungal specimens representing 94 *Xylodon* taxa, 15 *Hyphodontia* J. Erikss taxa, five *Lyomyces* P. Karst taxa, and four *Kneiffiella* taxa. The average SD of split frequencies in BI analyses is 0.020943 (BI), and the effective sample size (ESS) for Bayesian analysis across the two runs is double the average ESS (avg ESS) = 1031.5. The phylogenetic tree (Fig. 6) reveals that the new species has a close relationship with *Xylodonbambusinus* C.L. Zhao & X. Ma, *X.fissuratus* C.L. Zhao, *X.subclavatus* (Yurchenko, H.X. Xiong and Sheng H. Wu) Riebesehl, Yurch. & Langer, *X.montanus* C.L. Zhao and *X.wenshanensis* K.Y. Luo & C.L. Zhao. Additionally, *Xylodongranulanoides* and *X.granulans* clustered together.

### ﻿Taxonomy

#### ﻿ Botryobasidium daweishanense

Taxon classificationFungiCantharellalesBotryobasidiaceae

J.L. Zhang, H.M. Zhou & C.L. Zhao sp. nov.

9104FC33-258B-540E-90AD-59E720D52954

856337

Figs 7 , 8

##### Diagnosis.

*Botryobasidiumdaweishanense* differs from *B.subcoronatum* by its araneose hymenial surface, smaller basidia, fusiform, cyanophilous, and wider basidiospores.

**Figure 7.**
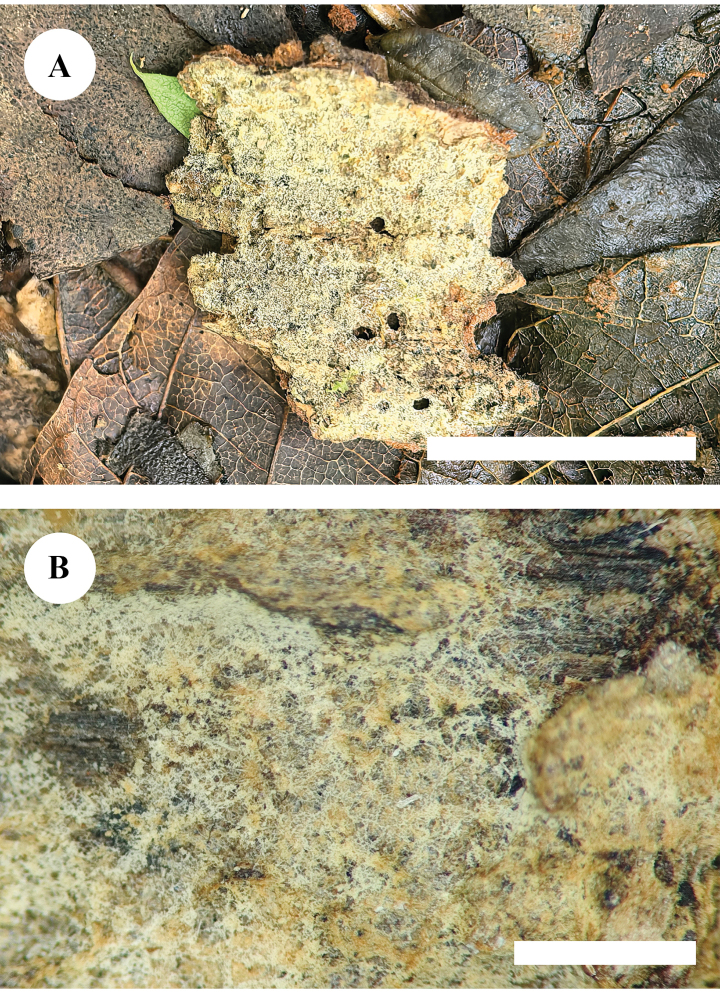
Basidiomata of *Botryobasidiumdaweishanense* (holotype, CLZhao 40062) **A** the front of the basidiomata **B** characteristic hymenophore. Scale bars: 1 cm (**A**); 1 mm (**B**).

##### Holotype.

China • Yunnan Province, Honghe, Pingbian County, Daweishan National Nature Reserve, 28°42'N, 114°11'E, evel. 1356 m asl., on fallen angiosperm branch, 1 August 2019, CLZhao 40062 (SWFC).

**Figure 8.**
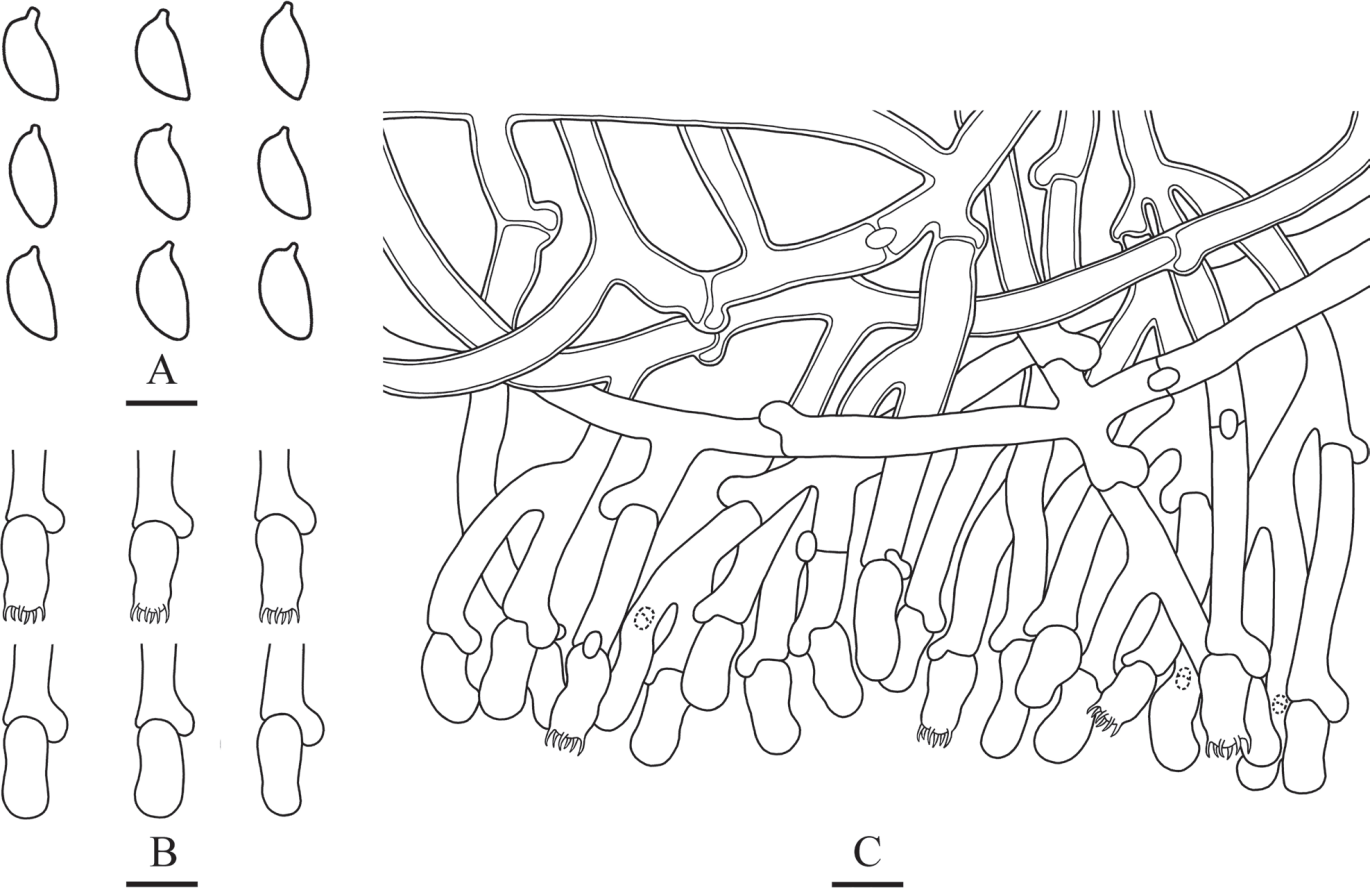
Microscopic structures of *Botryobasidiumdaweishanense* (holotype, CLZhao 40062) **A** basidiospores **B** basidia and basidioles **C** a section of hymenium. Scale bars: 5 μm (**A**); 10 μm (**B, C**); 10 × 100 oil.

##### Etymology.

*Daweishanense* (Lat.) refers to the type location “Daweishan National Nature Reserve,” China.

##### Description.

***Basidiomata*.** Annual, resupinate, coriaceous, without odor or taste when fresh, up to 1.6 cm long, 1.5 cm wide, and 100 μm thick. Hymenial surface araneose, cream when fresh, straw-yellow to yellowish when dry. Sterile margin thin, indistinct, slightly yellowish, up to 0.5 mm.

***Hyphal system*.** Monomitic; generative hyphae with clamp connections, hyaline, thin to slightly thick-walled, frequently branched, interwoven, 5.0–8.0 μm in diam, IKI–, CB+; tissues unchanged in KOH.

***Hymenium*.** Cystidia and cystidoles absent. Basidia barred, slightly sinuous, with six short sterigmata and a basal clamp connection, 11.5–18.0 × 5.0–8.0 μm; basidioles dominant, in shape similar to basidia, but slightly smaller. ***Basidiospores*.** Fusiform, hyaline, thin-walled, smooth, IKI–, CB+, (6.0–)6.1–7.3 × (3.1–)3.3–3.9(–4.1) μm, L = 6.65 μm, W = 3.64 μm, Q = 1.81–1.85 (n = 60/2).

##### Additional specimens examined.

China • Yunnan Province, Honghe, Pingbian County, Daweishan National Nature Reserve, 28°42'N, 114°11'E, evel. 1356 m asl., on fallen angiosperm branch, 1 August 2019, CLZhao 40061 (SWFC).

#### ﻿ Inonotus subglobisporum

Taxon classificationFungiHymenochaetalesHymenochaetaceae

J.L. Zhang, H.M. Zhou & C.L. Zhao sp. nov.

9676140F-2969-5885-8DC0-E88158395C85

856340

Figs 9 , 10

##### Diagnosis.

*Inonotussubglobisporum* differs from *I.radiata* by its perennial basidiomata, laterally stipitate, polygon pores, tapered, dark brown, thick-walled setae, and subglobose, thick-walled, hyaline basidiospores.

**Figure 9.**
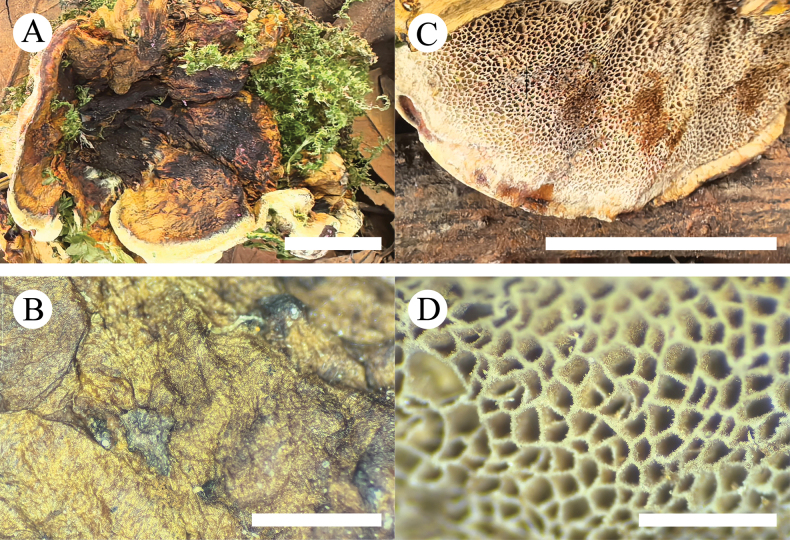
Basidiomata of *Inonotussubglobisporum* (holotype, CLZhao 8765) **A, B** the front of the basidiomata **C, D** characteristic hymenophore. Scale bars: 1 cm (**A, C**); 1 mm (**B, D**).

##### Holotype.

China • Yunnan Province, Puer, Jingdong County, Ailaoshan National Nature Reserve, 23°42'N, 101°52'E, evel. 2450 m asl., on fallen angiosperm trunk, 25 August 2018, CLZhao 8765 (SWFC).

**Figure 10.**
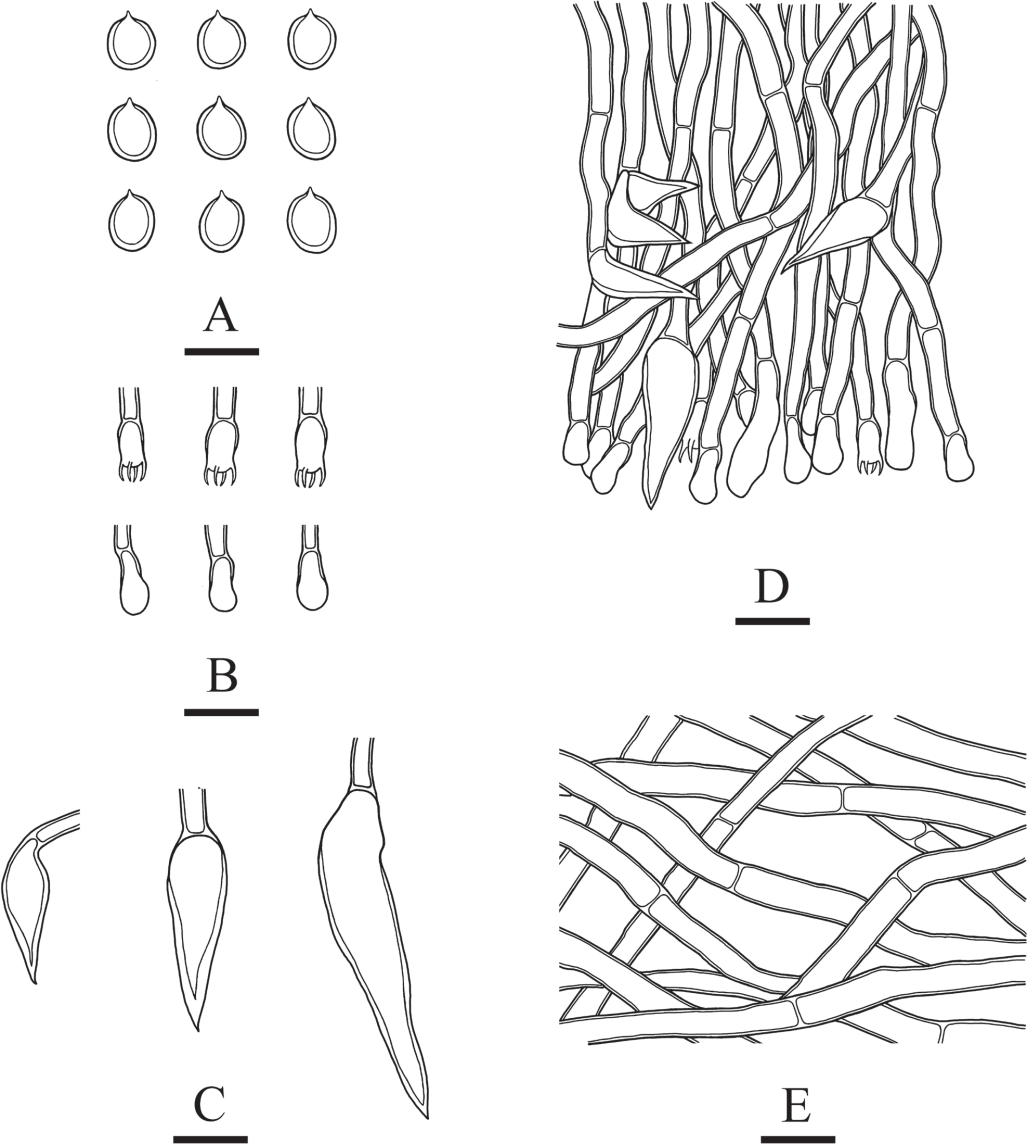
Microscopic structures of *Inonotussubglobisporum* (holotype, CLZhao 8765) **A** basidiospores **B** basidia and basidioles **C** setae **D** hymenium and hyphae from trama **E** hyphae from context. Scale bars: 5 μm (**A**); 10 μm (**B–E**); 10 × 100 oil.

##### Etymology.

*Subglobisporum* (Lat.) refers to the subglobose basidiospores.

##### Description.

***Basidiomata*.** Perennial, laterally stipitate, solitary, without odor or taste when fresh. Pilei fan-shaped, cortical to cork, extend up to 1.3 cm long, 2.2 cm wide, and 6 mm thick in diam at the base. Pileal surface honey-yellow to fuscous when fresh, vinaceous brown to fuscous when dry. Pore surface cream, when fresh, becomes cream to fawn when dry; pores polygonal, 4–6 per mm. Context cinnamon-buff when dry, cork, up to 5 mm thick. Tubes cream to fawn when dry, cork, up to 1 mm long. Stipe with the same color as pores, up to 23 mm long and 15 mm in diameter when dry.

***Hyphal structure*.** Monomitic; generative hyphae, simple-septate, slightly thick-walled, frequently branched, interwoven. Generative hyphae in the tube frequent, brownish, slightly thick-walled, easily collapsing, 2.5–5.0 µm in diam. Generative hyphae in the context frequent, brown, slightly thick-walled, 3.0–7.0 µm in diam, IKI–, CB–; tissues brownish in KOH. ***Context*.** Setae numerous, tapered, dark brown, thick-walled, strongly encrusted in the surface, and almost entirely buried, 12.0–69.5 × 4.0–11.0 μm, cystidoles absent. Basidia clavate, with four short sterigmata and a basal simple-septate, 5.5–13.0 × 2.5–6.5 μm; basidioles in shape similar to basidia, but slightly smaller. ***Basidiospores*.** Subglobose, hyaline, thick-walled, smooth, IKI–, CB+, (3.5–)3.6–4.3(–4.4) × (2.6–)2.8–3.5(–3.6) μm, L = 3.99 μm, W = 3.20 μm, Q = 1.22–1.27 (n = 120/4).

##### Other specimens examined.

China • Yunnan Province, Puer, Jingdong County, Ailaoshan National Nature Reserve, 23°42'N, 101°52'E, evel. 2450 m asl., on fallen angiosperm trunk, 23 August 2018, CLZhao 8331; CLZhao 8387; CLZhao 8433; CLZhao 8543; 24 August 2018, CLZhao 8500; 25 August 2018, CLZhao 8678; CLZhao 8737; CLZhao 8789 (SWFC).

#### ﻿ Kneiffiella bubalina

Taxon classificationFungiHymenochaetalesHyphodontiaceae

J.L. Zhang, H.M. Zhou & C.L. Zhao sp. nov.

123371FA-83FE-50F0-8AE4-553EF3F4D851

856342

Figs 11 , 12

##### Diagnosis.

*Kneiffiellabubalina* differs from *K.subalutacea* by its cream basidiomata and cylindrical to slightly allantoid basidiospores.

**Figure 11.**
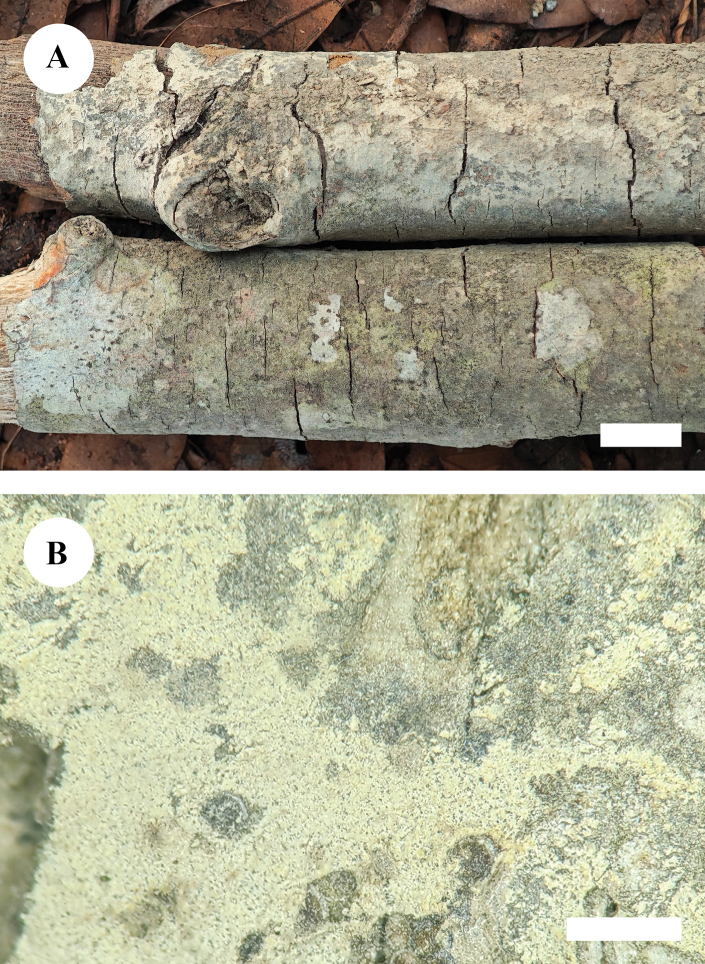
Basidiomata of *Kneiffiellabubalina* (holotype, CLZhao 15708) **A** the front of the basidiomata **B** characteristic hymenophore. Scale bars: 1 cm (**A**); 1 mm (**B**).

##### Holotype.

China • Yunnan Province, Wenshan, Xichou County, Jiguanshan Forestry Park, 23°53'N, 104°82'E, evel. 1730 m asl., on fallen angiosperm branch, 22 July 2019, CLZhao 15708 (SWFC).

**Figure 12.**
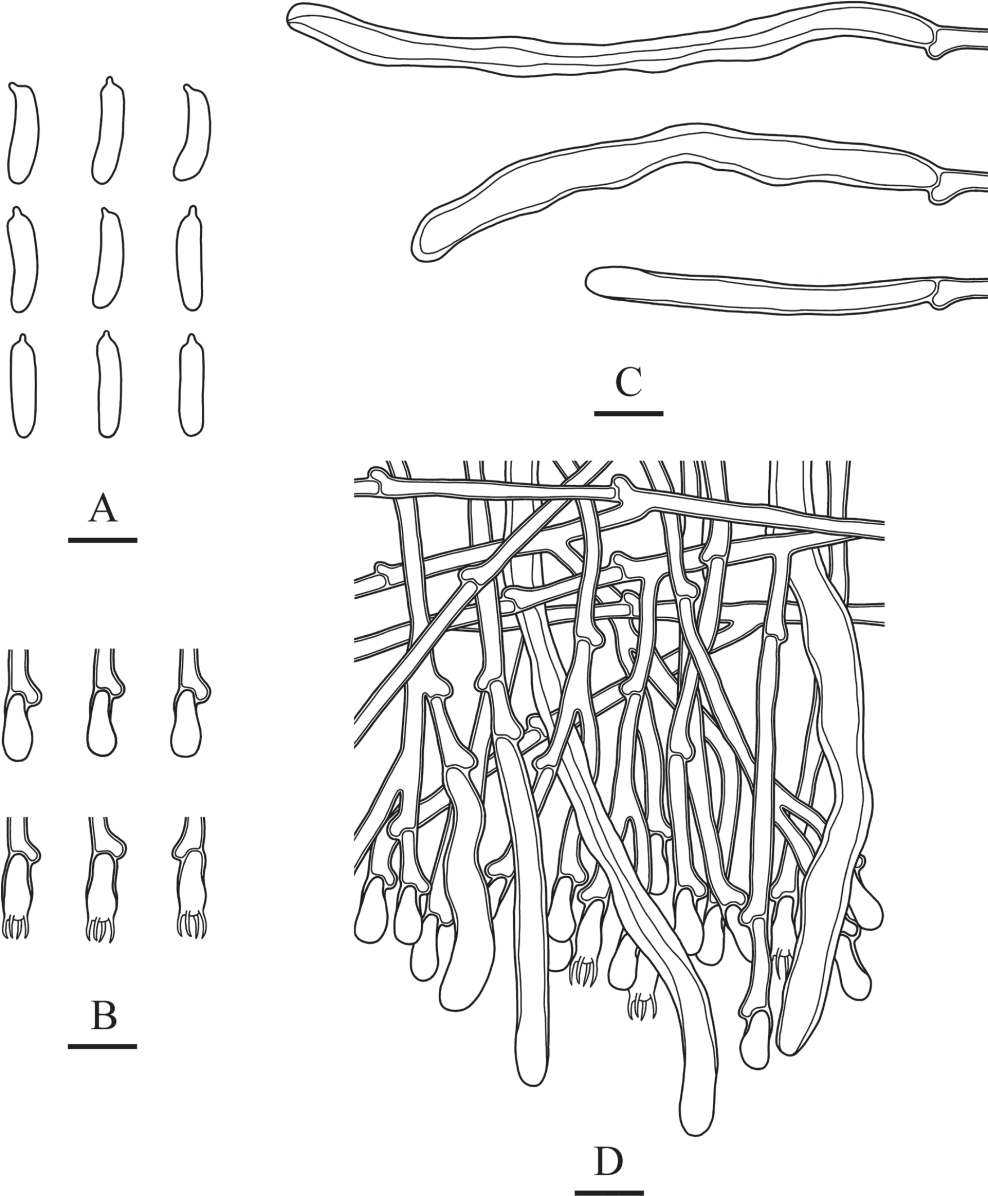
Microscopic structures of *Kneiffiellabubalina* (holotype, CLZhao 15708) **A** basidiospores **B** basidia and basidioles **C** cystidia **D** a section of hymenium. Scale bars: 5 μm (**A**); 10 μm (**B–D**); 10 × 100 oil.

##### Etymology.

*Bubalina* (Lat.) refers to its buff-colored hymenial surface.

##### Description.

***Basidiomata*.** Annual, resupinate, smooth, membranous, without odor or taste when fresh, up to 1.6 cm long, 1.1 cm wide, and 0.1–0.2 mm thick. Hymenial surface araneose, white to cream when fresh, buff when dry. Sterile margin thin, indistinct, slightly buff, up to 1 mm.

***Hyphal system*.** Monomitic; generative hyphae with clamp connections, slightly thick-walled, frequently branched, interwoven, IKI–, CB–, 2.5–3.5 μm in diam; tissues unchanged in KOH.

***Hymenium*.** Cystidia numerous, tubular, rising from subiculum with a basal clamp connection, hyaline, thick-walled except in the apical part, smooth, 103.5–162.5 × 6.0–8.0 μm; cystidioles absent. Basidia club-shaped, slight constriction in the middle part, with four sterigmata and a basal clamp connection, 10.0–14.0 × 4.5–5.0 μm; basidioles dominant, in shape similar to basidia, but slightly smaller. ***Basidiospores*.** Cylindrical to slightly allantoid, slightly narrower in apical part, hyaline, thin-walled, smooth, IKI–, CB–, 8.0–8.9(–9.1) × (1.7–)1.8–2.3(–2.6) μm, L = 8.41 μm, W = 2.03 μm, Q = 4.15 (n = 30/1).

#### ﻿ Xylodon granulanoides

Taxon classificationFungiHymenochaetalesSchizoporaceae

J.L. Zhang, H.M. Zhou & C.L. Zhao sp. nov.

6A26C887-B1E1-5969-B95B-1A49121DD126

856343

Figs 13 , 14

##### Diagnosis.

*Xylodongranulanoides* differs from *X.granulans*. by its varied cystidia and broadly ellipsoid, thick-walled basidiospores measuring 4.7–5.3 × 3.6–4.1 μm.

**Figure 13.**
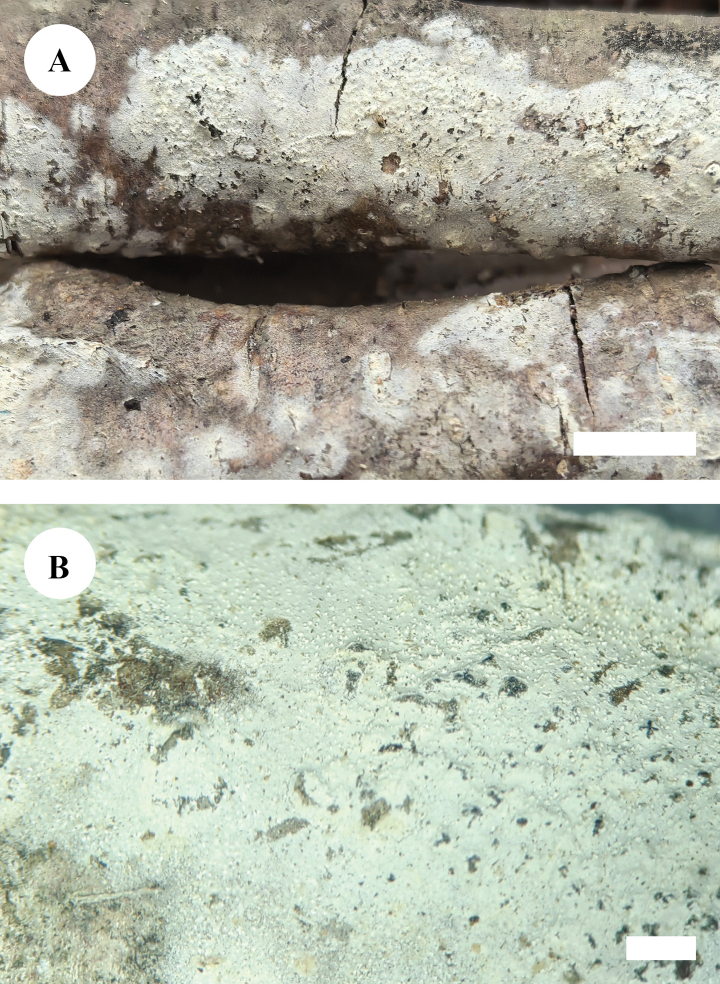
Basidiomata of *Xylodongranulanoides* (holotype, CLZhao 17253) **A** the front of the basidiomata **B** characteristic hymenophore. Scale bars: 1 cm (**A**); 1 mm (**B**).

##### Holotype.

China • Yunnan Province, Wenshan, Pingbian County, Wenshan National Nature Reserve, 23°22'N, 103°93'E, evel. 1753 m asl., on fallen angiosperm branch, 28 July 2019, CLZhao 17253 (SWFC).

**Figure 14.**
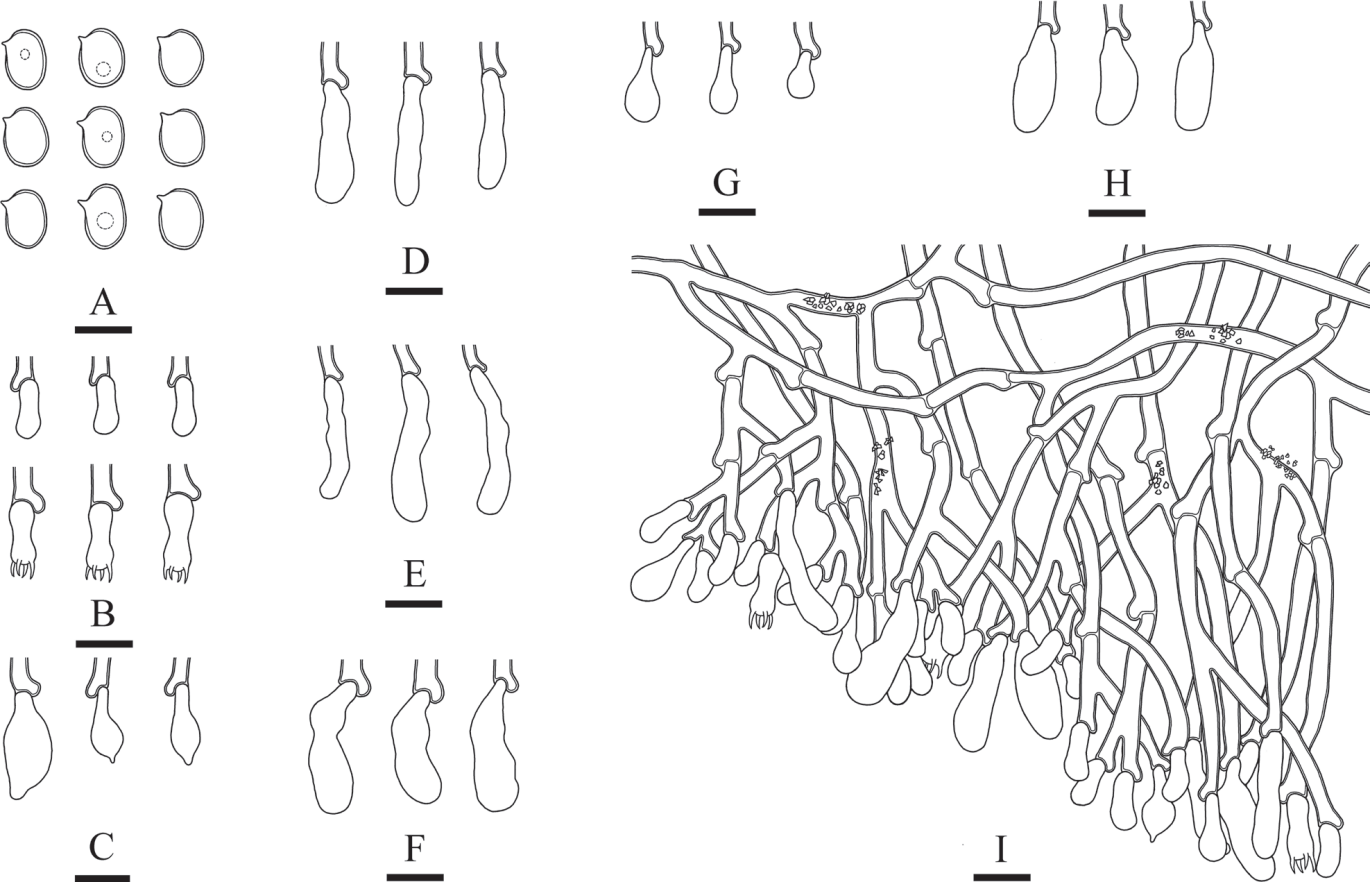
Microscopic structures of *Xylodongranulanoides* (holotype, CLZhao 17253) **A** basidiospores **B** basidia and basidioles **C** cystidioles **D–H** cystidia **I** a section of hymenium. Scale bars: 5 μm (**A**); 10 μm (**B–I**); 10 × 100 oil.

##### Etymology.

*Granulanoides* (Lat.) refers to the new species’ resemblance to *Xylodongranulans*.

##### Description.

***Basidiomata*.** Annual, resupinate, adnate, membranous, without odor or taste when fresh, brittle when dry, up to 5.3 cm long, 2.5 cm wide, and 0.1 mm thick. Hymenial surfaces grandinioid, white to cream when fresh, cream to slightly buff upon drying. Sterile margin thin, indistinct, slightly cream, up to 1 mm.

***Hyphal system*.** Monomitic; generative hyphae with clamp connections slightly encrusted with crystals amongst generative hyphae, hyaline, slightly thick-walled, frequently branched, interwoven, 2.5–3.5 μm in diam, IKI–, CB–; tissues unchanged in KOH.

***Hymenium*.** Cystidia numerous, subclavate to cylindrical, or slightly subcapitate, hyaline, thin-walled, 9.5–29.0 × 4.0–9.5 μm; cystidioles are present, subcapitate, hyaline, thin-walled, 13.5–21.0 × 5.0–9.0 μm. Basidia subcylindrical to clavate, hyaline, thin-walled, with four sterigmata and a basal clamp connection, 11.0–16.0 × 4.0–5.5 µm; basidioles dominant, in shape similar to basidia, but slightly smaller. ***Basidiospores*.** Broadly ellipsoid, part has a large drop of oil, hyaline, slightly thick-walled, smooth, IKI–, CB–, (4.6–)4.7–5.3(–5.3) × (3.4–)3.6–4.1(–4.2) μm, L = 4.92 μm, W = 3.90 μm, Q = 1.26 (n = 30/1).

#### ﻿ Xylodon granulans

Taxon classificationFungiHymenochaetalesSchizoporaceae

J.L. Zhang, H.M. Zhou & C.L. Zhao sp. nov.

3955F00F-67DC-5235-BE5D-947988B06E48

856344

Figs 15 , 16

##### Diagnosis.

*Xylodongranulans* differs from *X.wenshanensis* by its broadly ellipsoid, thin-walled basidiospores measuring 3.8–4.2 × 2.9–3.3 μm.

**Figure 15.**
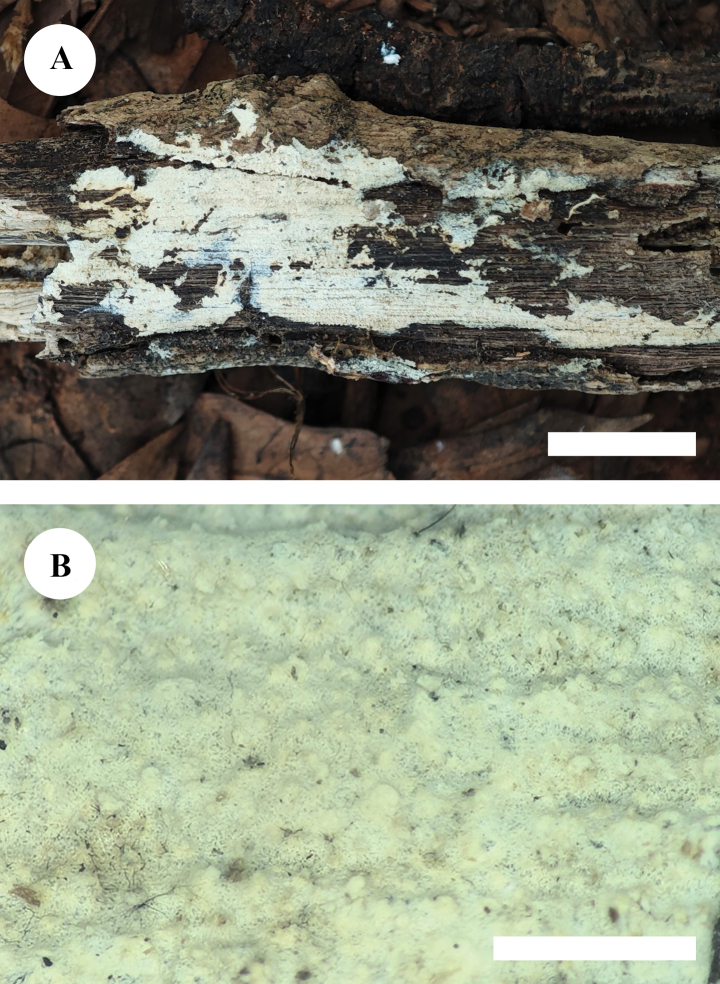
Basidiomata of *Xylodongranulans* (holotype, CLZhao 17866) **A** the front of the basidiomata **B** characteristic hymenophore. Scale bars: 1 cm (**A**); 1 mm (**B**).

##### Holotype.

China • Yunnan Province, Honghe, Pingbian County, Daweishan National Nature Reserve, 28°42'N, 114°11'E, evel. 1356 m asl. on fallen angiosperm branch, 1 August 2019, CLZhao 17866 (SWFC).

**Figure 16.**
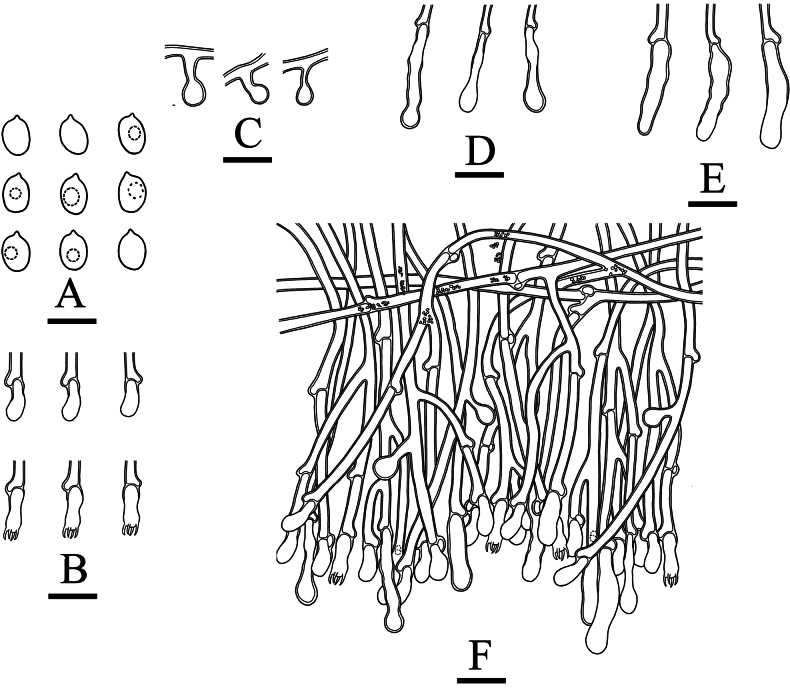
Microscopic structures of *Xylodongranulans* (holotype, CLZhao 17866) **A** basidiospores **B** basidia and basidioles **C–E** cystidia **F** a section of hymenium. Scale bars: 5 μm (**A**); 10 μm (**B–F**); 10 × 100 oil.

##### Etymology.

*Granulans* (Lat.) refers to the granulated hymenial surface.

##### Description.

***Basidiomata*.** Basidiomata annual, resupinate, adnate, membranous, without odor or taste when fresh, up to 2.4 cm long, 1.3 cm wide, and 50–70 μm thick. Hymenial surface grandinioid, cream when fresh, white to slightly cream when dry. Sterile margin thin, indistinct, slightly cream, up to 1 mm.

***Hyphal system*.** Monomitic; generative hyphae with clamp connections slightly encrusted with crystals amongst generative hyphae, hyaline, slightly thick-walled, frequently branched, interwoven, 2.0–2.5 μm in diam, IKI–, CB–; tissues unchanged in KOH.

***Hymenium*.** Cystidia of two types: (1) Capitate cystidia in hymenium and subiculum, hyaline, slightly thick-walled, smooth, slightly constricted at the neck, with a globose head, 4.5–23.5 × 3.0–4.5 μm; (2) Clavate cystidia, slightly sinuous, hyaline, slightly thick-walled, smooth, 13.0–24.0 × 4.0–5.0 μm. Basidia clavate, slightly sinuous, with four sterigmata and a basal clamp connection, 9.0–12.0 × 3.5–4.5 μm; basidioles dominant, in shape similar to basidia, but slightly smaller. ***Basidiospores*.** Broadly ellipsoid, some of them with an oily drop, hyaline, thin-walled, smooth, IKI–, CB–, (3.7–)3.8–4.2(–4.3) × (2.8–)2.9–3.3(–3.4) μm, L = 4.03 μm, W = 3.12 μm, Q = 1.29–1.29 (n = 60/2).

##### Other specimen examined.

China • Yunnan Province, Honghe, Pingbian County, Daweishan National Nature Reserve, 28°42'N, 114°11'E, evel. 1356 m asl., on fallen angiosperm branch, 1 August 2019, CLZhao 17804 (SWFC).

## ﻿Discussion

Numerous wood-inhabiting fungal taxa have recently been identified over the last few years (Cui et al. 2019; Guan et al. 2023; Zhao et al. 2023b; Luo et al. 2024; Zhou et al. 2024b). The most recent taxonomic framework recognizes 14 families within Hymenochaetales (Wang et al. 2023; He et al. 2024; Wang and Zhou 2024). To further investigate the wood inhabiting fungal diversity, collections representing five new species *viz. Inonotussubglobisporum*, *Kneiffiellabubalina*, *Xylodongranulanoides*, *X.granulans* (Hymenochaetales), and *Botryobasidiumdaweishanense* (Cantharellales) from Yunnan Province were collected and are introduced based on a combination of morphological features and molecular evidence.

*Botryobasidiumdaweishanense* is characterized by an araneose hymenial surface, generative hyphae with clamp connections, subcylindrical basidia (11.5–18.0 × 5.0–8.0 μm), and fusiform, cyanophilous basidiospores (6.1–7.3 × 3.3–3.9 μm). In several previous studies, molecular data have confirmed phylogenetic relationships showing that the genus *Botryobasidium* is nested within the cantharelloid clade and grouped with related genera such as *Cantharellus* Lam., *Clavulina* J. Schröt, *Craterellus* Pers, and *Hydnum* L. (Moncalvo et al. 2006; Bernicchia and Gorjón 2010). Macromorphologically, species of *Botryobasidium* are often mistaken for certain genera, such as *Ceratobasidium* D.P. Rogers, *Sistotrema* Fr., and *Tulasnella* J. Schröt (Donk 1956; Oberwinkler 1982). However, *Botryobasidium* is distinguished from these genera by the absence of epibasidia, sturdy and long sterigmata, and oily inclusions (Kotiranta and Saarenoksa 2005; Gorjón and Hallenberg 2008; Oberwinkler et al. 2017; Zhou et al. 2024c). The phylogram created based on inferences from the ITS+nLSU data in the present study aligns with previous research. According to the phylogram (Fig. 1), the new species of *Botryobasidiumdaweishanense* was grouped into the genus *Botryobasidium* (Botryobasidiaceae). Phylogenetic analysis of the ITS system (Fig. 2) revealed that the new species *Botryobasidiumdaweishanense* is grouped with three taxa: *B.intertextum*, *B.leptocystidiatum*, and *B.subcoronatum*. However, morphologically, *Botryobasidiumintertextum* differs macroscopically from *B.daweishanense* in being initially thin, hypochnoid, and white and later displaying pellicular and yellowish hymenial surfaces. In contrast, the hymenial surface of *B.daweishanense* is araneose and exhibits a straw-yellow to cream color when dry. In addition, at the micro level, *Botryobasidiumintertextum* is distinguishable from *B.daweishanense* by its larger basidiospores (7.0–9.5 × 1.8–2.81 μm) and basidia (15–21 × 5–6.5 μm; Kotiranta and Saarenoksa 1990). *Botryobasidiumleptocystidiatum* and *B.daweishanense* both display an arachnoid hymenial surface macroscopically; however, *B.daweishanense* appears cream-colored when fresh and straw-yellow to yellowish when dry, whereas *B.leptocystidiatum* is grayish-white to smoky gray when fresh and grayish-white to ivory when dry. Additionally, microscopically, *B.leptocystidiatum* features tubular cystidia, 6–7 sterigmata, and smaller basidia (10.5–15 × 7–8 μm) and longer basidiospores (6.5–7.8 × 2.9–3.7 μm) than *B.daweishanense* (Zhou et al. 2024c). *Botryobasidiumsubcoronatum* is distinguished from *B.daweishanense* by the former being thin, floccose to hypochnoid, and whitish at first, followed by having a yellowish to ochraceous hymenial surface. On a microscopic level, the species *Botryobasidiumsubcoronatum* has relatively larger basidia (20–25 × 7–9 µm) and narrower basidiospores (6–7.5 × 2.5–3 µm; Eriksson and Ryvarden 1973).

*Inonotussubglobisporum* is characterized by perennial, laterally stipitate basidiomata; tapered, dark brown, thick-walled setae (12.0–69.5 × 4.0–11.0 μm); and subglobose, cyanophilous basidiospores (3.6–4.3 × 2.8–3.5 μm). In the phylogenetic tree of Wu et al. (2022), species of *Inonotus* formed a monophyletic clade; however, *Inonotus* may still be a polyphyletic genus because most species within the genus have not been phylogenetically analysed. Wagner and Fischer also regarded *Inonotus* as a polyphyletic group (Wagner and Fischer 2002; Lin et al. 2023). In the present study, the phylogenetic analysis of the ITS system (Fig. 4) was consistent with previous reports and revealed that the species *Inonotussubglobisporum* was a sister to *I.radiatus*. However, morphologically, the species *Inonotusradiatus* differs from *I.subglobisporum* in terms of its annual basidiomata, which are typically in imbricate clusters; hooked setae; and ellipsoid, hyaline to pale yellowish basidiospores (3.8–5 × 2.6–3.5 μm; Dai 2010).

*Kneiffiellabubalina* is characterized by cream-colored basidiomata, slightly thick-walled generative hyphae, and cylindrical to slightly allantoid basidiospores (8.0–8.9 × 1.8–2.3 μm). Riebesehl and Langer (2017) showed the monophyly of *Kneiffiella* species by inferring ITS sequences using Bayesian analysis. Wang et al. (2021) classified *Kneiffiella* as belonging to the family Chaetoporellaceae (Riebesehl and Langer 2017; Wang et al. 2021; Langer et al. 2022). Based on a phylogenetic analysis of the ITS + nLSU system (Fig. 3), we determined that the genus *Kneiffiella* is nested in the Chaetoporellaceae clade. Phylogenetic analysis of the ITS system (Fig. 5) revealed that the new species, *Kneiffiellabubalina*, is a sister to *K.subalutacea*. Morphologically, *Kneiffiellasubalutacea* resembles *K.bubalina* because of its smooth hymenial surface, tubular, obtuse apical part, and thick-walled cystidia. However, *Kneiffiellasubalutacea* can be distinguished from *K.bubalina* by its yellowish basidiomata and allantoid, smooth, thin-walled basidiospores (6–8 × 1.5–2.0 μm; Eriksson and Ryvarden 1976).

*Xylodongranulanoides* is characterized by grandinioid hymenial surfaces, varied cystidia, and broadly ellipsoid, thick-walled basidiospores (4.7–5.3 × 3.6–4.1 μm). *Xylodongranulans* is characterized by grandinioid hymenial surfaces, capitate cystidia and clavate cystidia, and broadly ellipsoid, thin-walled basidiospores (3.8–4.2 × 2.9–3.3 μm). In several previous phylogenetic studies based on multiple loci in the family Schizoporaceae, three genera, *Fasciodontia* Yurchenko & Riebesehl, *Lyomyces*, and *Xylodon*, were located in this family (Wang et al. 2021; Yuan and Zhao 2024). The present study’s phylogram inferred from the ITS+nLSU data (Fig. 3) shows that two new species, *Xylodongranulanoides* and *X.granulans*, are grouped within the family Schizoporaceae. Based on the ITS topology (Fig. 6), these two new species are closely clustered with five other species: *Xylodonbambusinus*, *X.fissuratus*, *X.montanus*, *X.subclavatus*, and *X.wenshanensis*; meanwhile, the taxon *X.granulans* is a sister to *X.granulanoides*. However, morphologically, *Xylodongranulans* differs from *X.granulanoides* in that *X.granulans* lacks cystidioles and exists as broadly ellipsoid, thin-walled basidiospores (3.8–4.2 × 2.9–3.3 μm). *Xylodonbambusinus* differs from *X.granulans* and *X.granulanoides* in its ceraceous basidiomata, fusiform cystidia and capitate cystidia, and broad, ellipsoid, thin-walled basidiospores (4–5.5 × 3–4 μm; Ma and Zhao 2021). *Xylodonfissuratus* and *X.montanus* differ from *X.granulans* and *X.granulanoides* in that *X.fissuratus* and *X.montanus* have only one cystidia. In contrast, *X.fissuratus* has capitate cystidia, a thin-walled hyphal structure, and ellipsoid, thin-walled basidiospores (4.0–5.0 × 3.0–4.0 µm; Guan et al. 2023). *Xylodonmontanus* has a smooth hymenial surface, moniliform cystidia, and ellipsoid to broad ellipsoid basidiospores (3.9–5.3 × 3.2–4.3 µm; Qu et al. 2022). *Xylodonsubclavatus* can be distinguished from *X.granulans* and *X.granulanoides* by its cracked, aculei wart-like to conical, blunt to acute hymenial surface, and four types of cystidia: subclavate, hyphoid, capitate to sucapitate, and moniliform (Yurchenko et al. 2013). *Xylodonwenshanensis* can be distinguished from *X.granulans* and *X.granulanoides* by its capitate cystidia, clavate cystidia, and ellipsoid, thin-walled basidiospores (3–5 × 2–3.5 μm; Luo et al. 2022).

The families Chaetoporellaceae, Hymenochaetaceae, and Schizoporaceae represent a well-studied group within Hymenochaetales (Luo and Zhao 2021; Langer et al. 2022; Cho et al. 2024; Freire and Soares 2024; Viner et al. 2024); however, the diversity of species in China remains poorly understood, particularly in the southwestern region. Additionally, the genus *Botryobasidium* has rarely been reported in recent years, particularly in southwestern China. Therefore, the species diversity of Cantharellales and Hymenochaetales in China, particularly in the subtropical and tropical regions, has not been sufficiently studied. This study contributes to our understanding of fungal diversity in these areas and underscores the urgent need for further fieldwork and molecular analyses to identify new taxa. Our findings confirm that fungal diversity is abundant in southwestern China.

## Supplementary Material

XML Treatment for Botryobasidium daweishanense

XML Treatment for Inonotus subglobisporum

XML Treatment for Kneiffiella bubalina

XML Treatment for Xylodon granulanoides

XML Treatment for Xylodon granulans

## ﻿Additional information

### Author ORCIDs

Jianling Zhang https://orcid.org/0009-0005-3268-7665

Zirui Gu https://orcid.org/0000-0003-1476-9040

Chunqin Zhou https://orcid.org/0009-0004-8117-1537

Hongmin Zhou https://orcid.org/0000-0002-0724-5815

### Data availability

All of the data that support the findings of this study are available in the main text.
